# The Tomato Prf Complex Is a Molecular Trap for Bacterial Effectors Based on Pto Transphosphorylation

**DOI:** 10.1371/journal.ppat.1003123

**Published:** 2013-01-31

**Authors:** Vardis Ntoukakis, Alexi L. Balmuth, Tatiana S. Mucyn, Jose R. Gutierrez, Alexandra M. E. Jones, John P. Rathjen

**Affiliations:** 1 School of Life Sciences, University of Warwick, Coventry, United Kingdom; 2 The Sainsbury Laboratory, Norwich Research Park, Colney, United Kingdom; The University of North Carolina at Chapel Hill, United States of America

## Abstract

The major virulence strategy of phytopathogenic bacteria is to secrete effector proteins into the host cell to target the immune machinery. AvrPto and AvrPtoB are two such effectors from *Pseudomonas syringae*, which disable an overlapping range of kinases in Arabidopsis and Tomato. Both effectors target surface-localized receptor-kinases to avoid bacterial recognition. In turn, tomato has evolved an intracellular effector-recognition complex composed of the NB-LRR protein Prf and the Pto kinase. Structural analyses have shown that the most important interaction surface for AvrPto and AvrPtoB is the Pto P+1 loop. AvrPto is an inhibitor of Pto kinase activity, but paradoxically, this kinase activity is a prerequisite for defense activation by AvrPto. Here using biochemical approaches we show that disruption of Pto P+1 loop stimulates phosphorylation *in trans*, which is possible because the Pto/Prf complex is oligomeric. Both P+1 loop disruption and transphosphorylation are necessary for signalling. Thus, effector perturbation of one kinase molecule in the complex activates another. Hence, the Pto/Prf complex is a sophisticated molecular trap for effectors that target protein kinases, an essential aspect of the pathogen's virulence strategy. The data presented here give a clear view of why bacterial virulence and host recognition mechanisms are so often related and how the slowly evolving host is able to keep pace with the faster-evolving pathogen.

## Author Summary

The bacteria *Pseudomonas syringae* is a pathogen of many crop species and one of the model pathogens for studying plant and bacterial arms race coevolution. In the current model, plants perceive bacteria pathogens via plasma membrane receptors, and recognition leads to the activation of general defenses. In turn, bacteria inject proteins called effectors into the plant cell to prevent the activation of immune responses. AvrPto and AvrPtoB are two such proteins that inhibit multiple plant kinases. The tomato plant has reacted to these effectors by the evolution of a cytoplasmic resistance complex. This complex is compromised of two proteins, Prf and Pto kinase, and is capable of recognizing the effector proteins. How the Pto kinase is able to avoid inhibition by the effector proteins is currently unknown. Our data shows how the tomato plant utilizes dimerization of resistance proteins to gain advantage over the faster evolving bacterial pathogen. Here we illustrate that oligomerisation of Prf brings into proximity two Pto kinases allowing them to avoid inhibition by the effectors by transphosphorylation and to activate immune responses.

## Introduction

Plant immunity is innate and relies on two levels of pathogen perception, underpinned by different recognition strategies [1]. The first level of perception occurs at the cell surface where plasma membrane receptors called pattern recognition receptors (PRRs) recognise and respond to conserve pathogen molecules called pathogen-associated molecular patterns (PAMPs). Classically, PAMPs are invariant molecules associated with particular taxonomic classes, and are very difficult for the pathogen to modify or discard [2]. Despite the overall conservation of PAMPs, recent studies have shown that in adapted pathogens their immunogenic epitopes are under positive selection to evade host immune detection [3], [4]. Nevertheless, so-called PAMP-triggered immunity (PTI) is highly effective and is usually overcome only by adapted pathogens that have evolved specific evasive strategies [5]. Chief amongst these strategies is secretion of protein virulence molecules called effectors, which target PRRs and other nodes of the immune system to abrogate transduction of the PAMP signal within the host, or to defeat host defences [6]. Examplars of this strategy are AvrPto and AvrPtoB, two unrelated effectors of the bacterial pathogen *Pseudomonas syringae*, which are secreted directly into the host cell where they target a PRR complex composed of the receptor kinases FLAGELLIN SENSING 2 (FLS2) and BRI1-ASSOCIATED KINASE 1 (BAK1) that forms after perception of bacterial flagellin by FLS2 [7]–[9]. Upon direct interaction between the receptor kinases and the bacterial effectors downstream signalling events are abolished, albeit through different mechanisms. AvrPtoB also targets the receptor kinase CHITIN ELICITOR RECEPTOR KINASE 1 (CERK1) [10].

Plants have evolved a second layer of perception based on the presence of pathogen effectors within the host cell. Host resistance (R) proteins containing a central nucleotide-binding (NB) motif, of the STAND class, and C-terminal leucine rich repeats (LRRs) recognise specific effectors directly or indirectly, and induce strong defences leading to hypersensitive cell death response (HR). The responses induced by PRRs and NB-LRRs respectively have not been separated experimentally, and it is thought that these classes of proteins simply represent different points of entry to the same defence network [11]. While direct activation of NB-LRRs is self-explanatory, the paradigm of indirect effector recognition is of particular interest and illustrates the ingenuity of evolution. Indirect recognition follows a principle in which an accessory protein forms a complex with the NB-LRR protein. In theory, the accessory protein is either a molecular target of the virulence effector, or a mimic of one [1]. Examples of such accessory proteins include Pto kinase, RIN4 and PBS1 kinase, for the NB-LRR proteins Prf, RPM1/RPS2 and RPS5, respectively [5].

The mechanisms by which accessory proteins communicate effector binding to the NB-LRR protein are unknown. For the described examples, each complex exists prior to effector interaction. Accessory proteins make contact with regions of the R proteins N-terminal to the NB domain [12]. Recently, NB-LRR protein oligomerisation has been described for the Prf [13], RPS5 [14], MLA10 [15], and L6 [16] proteins, and this also occurs through N-terminal sequences. However, the significance of oligomerisation for plant immunity is not yet clear [17].

The Pto/Prf complex recognises both the AvrPto and AvrPtoB effectors. The recognition event occurs through the accessory protein kinase Pto [18]. Prf oligomerises through a novel N-terminal (N-term) domain, which also coordinates binding of Pto-like kinases, thus bringing them into proximity [13]. Although AvrPto and AvrPtoB are not related structurally, both interact with Pto predominately via the kinase P+1 loop [19]–[22]. The P +1 loop normally positions the peptide substrate within the catalytic cleft for phosphorylation. Interestingly, mutations within this loop lead to a constitutive gain-of-function (CGF) phenotype of effector-independent HR [19]. In addition, P+1 loop mutations abrogate Pto kinase activity, and activate Prf-dependent signalling [20], [23]. While Pto requires kinase activity for effector-dependent activation, it is dispensable for CGF forms [20]. Overall, the role of kinase activity in control of Prf signalling is not understood but is of critical importance.

Here we show that Pto molecules transphosphorylate each other, but only when in complex with Prf, and only under conditions of complex activation. Pto was doubly phosphorylated within the kinase activation segment, and this was necessary but not sufficient for signalling. Full activation of the complex required additional disturbance of the P+1 loop, by mutation or by interaction with the effector. We derive a model for activation of the complex by effectors, and show how the oligomeric arrangement of the complex provides a trap for the effector that is very difficult for the pathogen to avoid.

## Results

### Effector activation of Pto is associated with a band shift that requires an intact Prf P-loop

**Figure 1:**
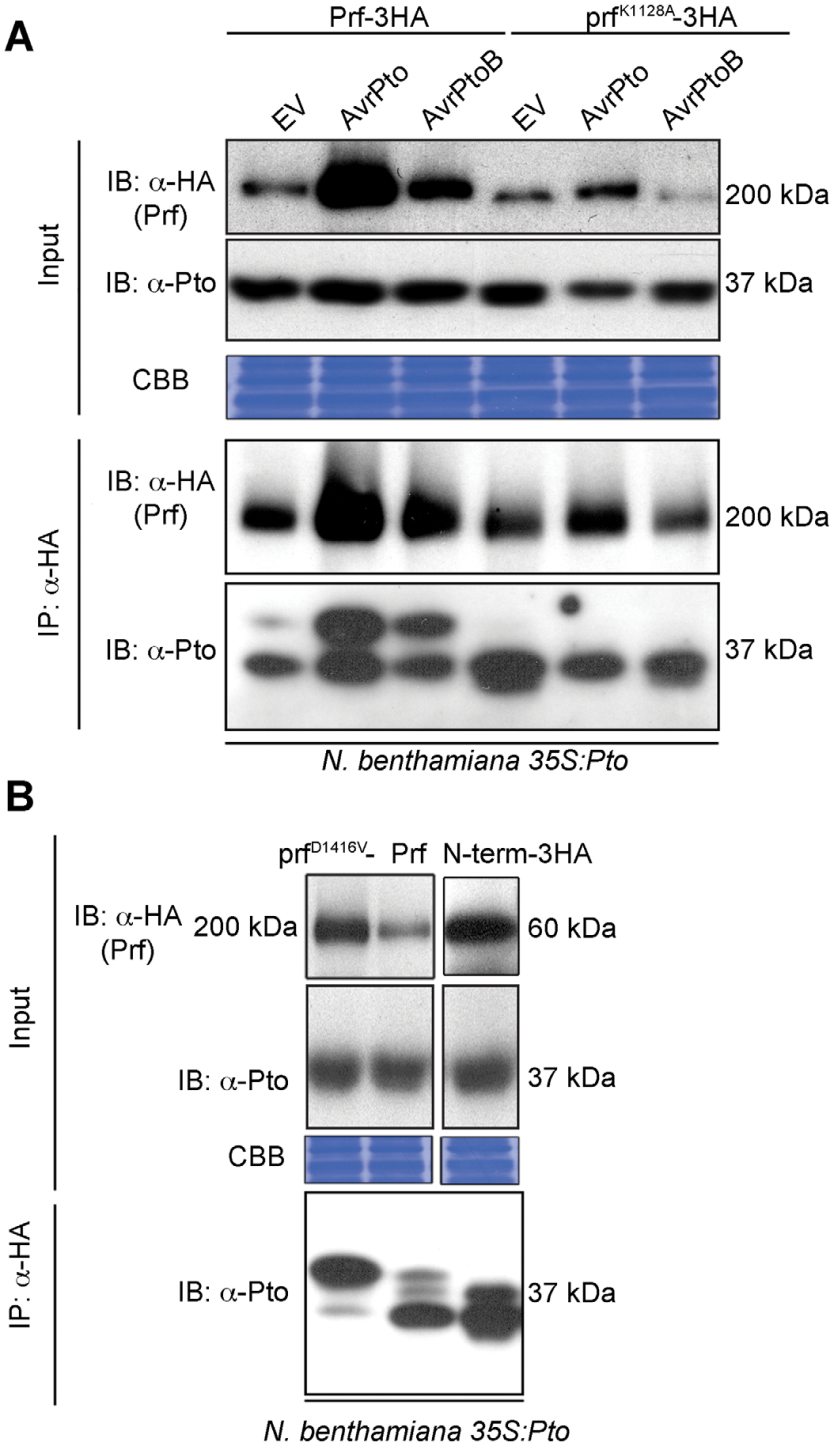
Phosphorylation of Pto upon activation of signalling. (**A**) Slow migration of Prf-associated Pto after effector recognition. The indicated Prf, AvrPto and AvrPtoB constructs were transiently expressed in stable transgenic 35S:*Pto N. benthamiana* plants. Three days post infiltration, Prf-3HA and prf^K1128A^-3HA proteins were immunoprecipitated (IP) using anti-HA antibodies. Immunoblots (IB) for Prf and Pto were performed with the antibodies indicated on the left. (**B**) A functional Prf molecule is required to generate the slow-migrating Pto form. The indicated Prf constructs were transiently expressed in stable transgenic 35S:*Pto N. benthamiana* plants. Three days post infiltration, Prf-3HA, prf^D1416V^-3HA and N-term-3HA (prf^1–546^-3HA) proteins were immunoprecipitated using anti-HA antibodies. Immunoblots were performed with the antibodies indicated on the left. Equal protein loading was verified by Coomassie Brilliant Blue (CBB) staining of the membranes. The experiment was repeated six times and typical results are shown.

To elucidate the role of Pto kinase activity within the Prf complex, we reconstituted this complex in the model species *Nicotiana benthamiana* by heterologous expression of its constituent components. In this system, co-expression of the tomato Pto and Prf proteins confers recognition of the effectors AvrPto and AvrPtoB leading to HR [24]. Although Pto kinase activity is required for its effector-dependent activation [19], previous experiments to detect activatory phosphorylation have not separated uncomplexed Pto from the small fraction that is bound to Prf [25]. To overcome this, we used *Agrobacterium tumefaciens* to express Prf transiently as a genetic fusion with three C-terminal haemagglutinin epitopes (Prf-3HA) in stable transgenic 35S:*Pto N. benthamiana* plants [24], which allowed us to purify Pto within the Prf complex by co-immunoprecipitation using anti-HA antibodies. We found that co-expression of AvrPto or AvrPtoB with the Pto/Prf complex correlated with the appearance of a slow-migrating form of Pto on SDS-PAGE (Figure 1A). A similar Pto band shift was observed previously [25] and its slight appearance in the empty vector (EV) control lacking effectors (three days post infiltration) was correlated with the ligand-independent signalling phenomenon in which overexpression of Pto and Prf induces HR (Figure S1A). This band shift of Pto was previously attributed to phosphorylation as it could be removed by treatment with phosphatase, but the phosphorylation sites were not identified [25]. Prf contains a central nucleotide-binding region conserved with plant and animal proteins of the NOD family [26]. Interestingly, mutation of a conserved residue within this region required for ATP binding, Lys-1128 (prf^K1128A^) [27], abolished the appearance of the slow migrating Pto band after co-expression with AvrPto or AvrPtoB (Figure 1A). This mutation also strongly diminished both the ligand-independent and effector-triggered HRs (Figures S1A and S1B). Taken together, these results demonstrate that AvrPto and AvrPtoB recognition by the Pto/Prf complex correlates with the appearance of a slow-migrating form of Pto and requires a functional Prf protein.

### The activation segment of Pto is doubly phosphorylated after activation by effectors

To investigate the observed band shift of Pto, we initially attempted to purify it from within the Prf complex by co-immunoprecipitation from *N. benthamiana* after heterologous expression of Prf -3HA, FLAG-tagged Pto, and effectors. After immunoprecipitation of Prf using anti-HA antibodies, we were unable to identify the putative Pto phosphorylation sites in these experiments for technical reasons. Subsequently, the total Pto protein comprising both the Prf-complexed and free forms were purified from *N. benthamiana*. Immunoprecipitated Pto was subjected to SDS-PAGE fractionation, in-gel tryptic digestion and mass spectrometric analysis. In three independent experiments, we identified peptides spanning over 80% of the Pto protein and found that residues Ser-198 and Thr-199 within the kinase activation segment peptide K/GTELDQTHLSTVVK were modified by phosphorylation (Table 1, Tables S1 and S2), a typical regulatory event in eukaryotic kinases [28]. Before effector recognition, Pto contained a single phosphorylation event attributed predominantly to Ser-198, as found previously [13], but some MS spectra also supported a single phosphorylation event on Thr-190 or Thr-199 (Figures S2 and S3).

**Table 1:** Double phosphorylation of Pto peptide 187–202 and 188–202 upon activation of signalling.

	Prf				prf^K1128A^	prf^D1416V^	prf^1–546^
Pto peptide 188–202	EV* (3 dpi)	AvrPto (2 dpi)	AvrPtoB (2 dpi)	EV (2 dpi) *control*	EV (2 dpi)	EV (2 dpi)	EV (2 dpi)
G[_p_T^190^]ELDQTHLSTVVK	0	0	0	0	0	0	1
GTELDQTHL[_p_S^198^]TVVK	2	4	3	9	16	13	20
GTELDQTHLS [_p_T^199^]VVK	2	3	3	8	3	3	7
**GTELDQTHL[_p_S^198^][_p_T^199^]VVK**	**2**	**7**	**2**	**0**	**0**	**1**	**0**
GTELDQTHLSTVVK	21	20	20	33	48	6	2
Pto peptide 187–202							
KGTELDQ[_p_T^195^]HLS TVVK	0	0	0	0	0	1	0
KGTELDQTHL[_p_S^198^]TVVK	14	14	11	14	16	8	5
KGTELDQTHLS [_p_T^199^]VVK	0	2	0	4	3	1	1
KG[_p_T^190^]ELDQTHLS[_p_T^199^]VVK	1	0	0	0	0	1	0
KGTELDQ[_p_T^195^]HLS [_p_T^199^]VVK	0	1	0	0	0	0	0
**KGTELDQTHL[_p_S^198^][_p_T^199^]VVK**	**1**	**5**	**3**	**0**	**0**	**4**	**0**
KGTELDQTHLSTVVK	11	32	8	14	26	6	5
peptides 187–202 and 188–202	54	88	50	83	112	47	41
percentage of peptides with [_p_S^198^] and [_p_T^199^]	5.5%	13.6%	10%	0%	0%	10.6%	0%
Pto Sequence Coverage	87%	75%	82%	76%	79%	38%	53%
**Signalling (HR)**	**yes** *	**yes**	**yes**	**no**	**no**	**yes**	**no**

Prf-3HA, prf^K1128A^ –3HA, prf^D1416V^ –3HA, N-term -3HA (prf^1–546^ –3HA), Pto-FLAG AvrPto and AvrPtoB were expressed transiently in *N. benthamiana* under the control of 35S promoter; the total amount of Pto-FLAG was immunoprecipitated using anti-FLAG antibodies. The number of peptides identified with 0, 1, and 2 phosphorylation events is indicated.

* ligand-independent HR.

Doubly phosphorylated peptides were identified under conditions of effector-activated signalling. The most frequently observed and strongly supported positions were Ser-198 and Thr-199 (Figures S2 and S3), although combinations of other sites were occasionally observed (Table 1, Tables S1 and S2). Despite the significantly fewer incidences of doubly phosphorylated peptides after AvrPtoB recognition in comparison to AvrPto recognition, the complete absence of the doubly phosphorylated peptides in the EV control (2 days post infiltration) is clear. Doubly phosphorylated peptides were also identified when signalling was activated in a ligand-independent manner (Table 1, EV 3 days post infiltration). Additional experiments were performed to elucidate the role of Prf activation in the appearance of doubly phosphorylated peptides.

### Pto complexed with gain-of-function prf^D1416V^ is doubly phosphorylated within its activation segment

**Figure 2:**
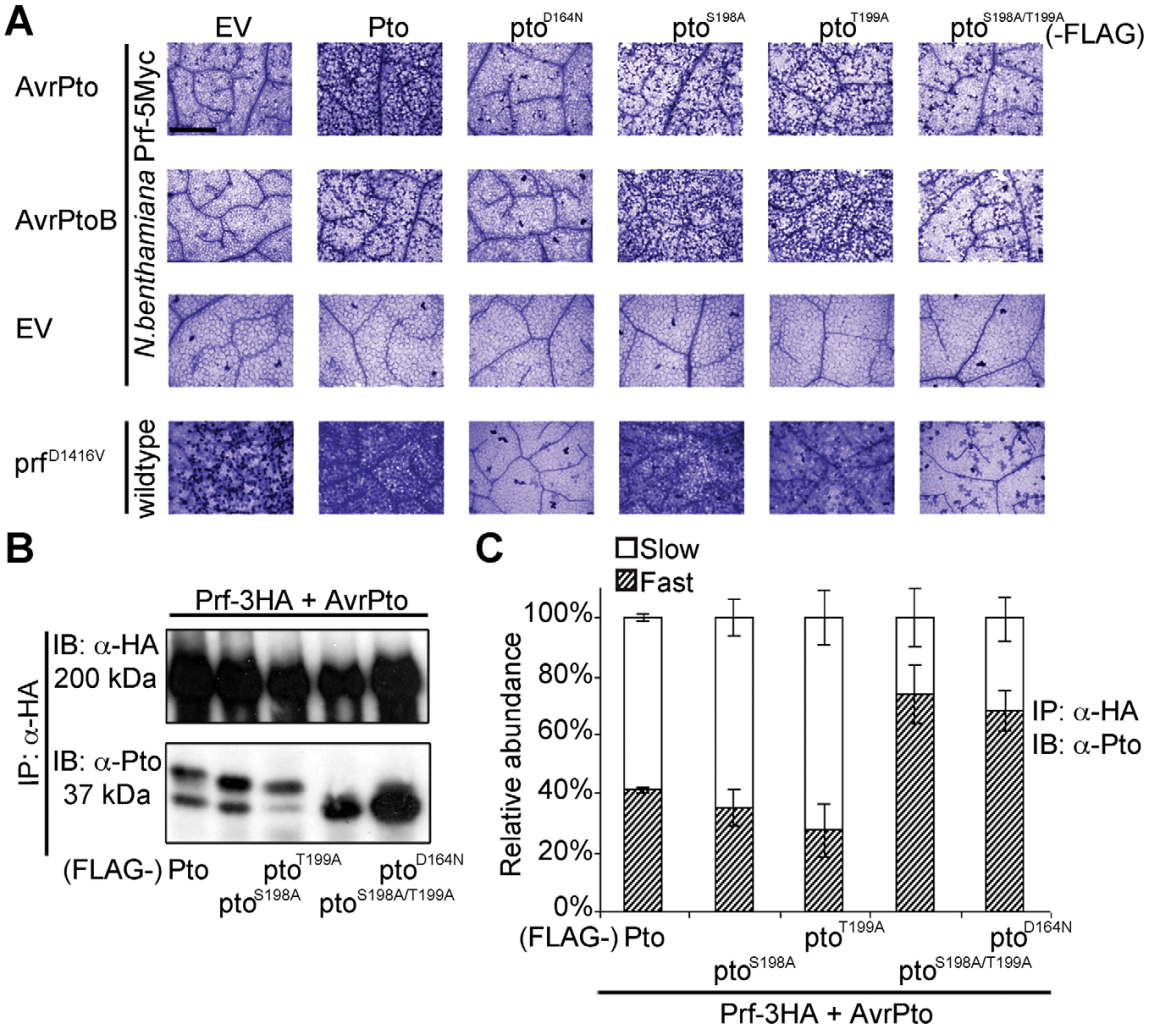
Phosphorylation on Pto residues S198 and T199 is required for signalling. (**A**) Trypan blue staining of cell death in *N. benthamiana* leaves. Pto-FLAG, pto mutant-FLAG, AvrPto, AvrPtoB, and prf^D1416V^-3HA constructs were transiently expressed in Pro_Prf_:*Prf-5Myc* or wild-type *N. benthamiana* as indicated and the tissue was stained 2 days post infiltration. The bar indicates 0.5 mm. Dead cells stain dark blue in this qualitative assay. Each row is derived from a single leaf, within which relative amounts of cell death were comparable, and is representative of six replicates. (**B**) The slow-migrating form of Pto requires kinase activity and double phosphorylation. Pto-FLAG, pto mutant-FLAG, AvrPto, and Prf-3HA constructs were transiently expressed in wild-type *N. benthamiana* as indicated, Prf-3HA was immunoprecipitated (IP) using anti-HA antibodies. Immunoblots (IB) were performed with the antibodies indicated on the left. (**C**) Quantification of the relative abundance of slow- and fast-migrating forms of Pto under elicitation conditions as described in B with Quantity One, Bio-Rad (adjusted volume = [CNT*mm2] data counts/mm^2^). Error bars are standard deviation of relative abundance between the same samples in independent immunoblots, probed with anti-Pto antibody.

Data presented above with the prf^K1128A^ loss of function mutation (Figure 1A) suggested that Prf activation influences Pto phosphorylation status. To explore this further, we created a mutation within the conserved MHD motif [29] of the NB domain that conferred an effector-independent CGF phenotype to Prf (Figure 2A) as previously described for Prf [30] and other NB-LRR proteins [27]. Using the same experimental system described above, we found that expression of the prf^D1416V^ CGF mutant greatly increased the proportion of the slow Pto form relative to co-expression of Pto with wild-type Prf, or with the isolated Prf N-term domain that constitutes the Pto binding moiety [25] (Figure 1B). In two independent experiments, the doubly phosphorylated K/GTELDQTHLSTVVK peptide was again identified under these conditions (Tables 1 and Table S2). Thus, active forms of the Prf complex are associated with double phosphorylation of Pto on this peptide. Interestingly, single phosphorylation of Ser-198 or Thr-199 was observed previously, among other *in vitro* phosphosites [31] that were not identified in these experiments with the exception of Ser-11 that was phosphorylated in an activation-independent manner.

### Phosphorylation of Pto at least one of Ser-198 or Thr-199 is required for downstream signalling

To determine the function of the identified Pto phosphorylation sites, we mutated them individually and in combination to non-phosphorylable alanine. We tested the ability of the substitution mutants pto^S198A^, pto^T199A^ and pto^S198A/T199A^ to cause cell death upon effector recognition by trypan blue staining. We used this qualitative assay of cell death and an image based estimation of cell death (Relative HR index) in all subsequent HR assays. Both single mutants induced cell death after AvrPto or AvrPtoB co-expression, comparable to wild-type Pto (Figure 2A and Figure S4) possibly by phosphorylation of the secondary sites Thr-190 and Thr-195 as previously observed (Table 1, Tables S1 and S2). In contrast, the double mutant pto^S198A/T199A^ and the kinase-dead mutant pto^D164N^ [19] were severely impaired in their ability to support signalling. The CGF activity of prf^D1416V^ was also strongly diminished by co-expression with pto^S198A/T199A^ or kinase-dead pto^D164N^. Furthermore, co-expression of the prf^K1128A^ mutant, that prevents the appearance of the slow migrating Pto band (Figure 1A), also diminished the CGF phenotype of prf^D1416V^ (Figure S5A) by direct interaction (Figure S5B). Thus, phosphorylation of at least one of these residues (Ser-198 or Thr-199) is required for full activation of the Prf complex. The ability of each mutant to support cell death was again tightly correlated with the presence of a slower migrating Pto band, and the absence of the slower band in the pto^S198A/T199A^ mutant indicates that phosphorylation of at least one of these sites is required for the band shift (Figure 2B and C).

### The pto^S198A/T199A^ double mutant is an active kinase

To assess if Ser-198 and Thr-199 are required for Pto kinase activity, we tested the mutants described above for autophosphorylation activity or the ability to transphosphorylate the substrates Pti1 [32] and AvrPtoB [33]. Wild-type Pto and the mutants pto^S198A^, pto^T199A^ and pto^S198A/T199A^ were active kinases. The estimated relative autophosphorylation activities of pto^S198A^, pto^T199A^ and pto^S198A/T199A^ were comparable to wild-type Pto (Table 2 and Figure S6). Most importantly, their relative transphosphorylation activities were not correlated with ability to signal. pto^T199A^ and pto^S198A/T199A^ were able to transphosphorylate AvrPtoB and Pti1^K96N^ at comparable levels *in vitro* (Figure S6) but pto^T199A^ induced a much stronger cell death *in vivo* after AvrPtoB recognition (Figure 2A). Furthermore, in contrast to the kinase inactive mutant pto^D164N^, the kinase active pto^S198A/T199A^ did not support cell death upon recognition of the E3 ligase mutant avrPtoB^F479A^ [33] (Figure S7A and B), further substantiating the notion that the kinase activity is not correlated with the ability to signal. Therefore, double phosphorylation of Pto activation segment including phosphorylation of at least one of Ser-198 and Thr-199 is the signalling determinant, not kinase activity *per se*.

**Table 2:** Summary of Pto signalling activities.

Pto variants	Pto	pto^D164N^	pto^S198A^	pto^T199A^	pto^S198A/T19A^
Autophosphorylation^1^	**+**	**−**	**+**	**+**	**+**
Transphosphorylation of AvrPtoB^1^	**+**	**−**	**+**	**+**	**+**
Transphosphorylation of pti1^K96N^	**+**	**−**	**+**	**+**	**+**
Signalling (AvrPto, AvrPtoB, prf^D1416V^)^2^	**+**	**−**	**+**	**+**	**−**
Signalling (avrPtoB^F479A^)^3^	**+**	**+**	**+**	**+**	**−**

1 From Figure S6.

2 From Figure 2A.

3 From Figure S7.

### The double phosphorylation event is due to transphosphorylation within the Pto/Prf complex

**Figure 3:**
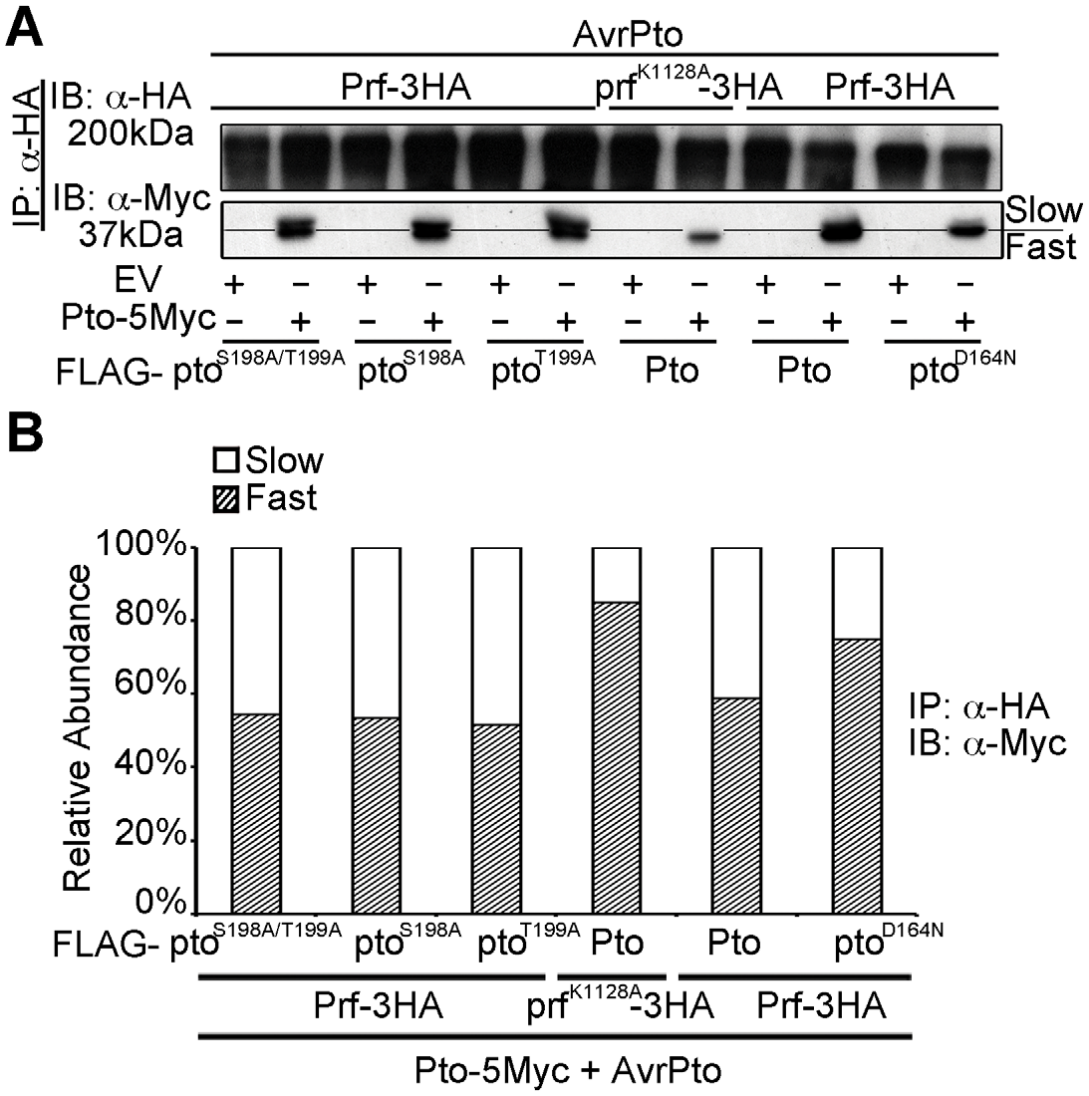
Transphosphorylation is required for Ser-198 and Thr-199 phosphorylation. (**A**) *In trans* inhibition of Pto S198 and T199 phosphorylation. The slower migrating from of Pto is suppressed *in trans* by prf^K1128A^ and pto^D164N^, but not pto^S189A/T199A^, pto^S189A^ and pto^T199A^. Pto-5Myc, Pto-FLAG, pto mutant-FLAG, AvrPto, Prf-3HA, prf^K1128A^-3HA constructs were transiently expressed in wild-type *N. benthamiana* as indicated, Prf-3HA and prf^K1128A^-3HA were immunoprecipitated (IP) using anti-HA antibodies. Immunoblots (IB) were performed with the antibodies indicated on the left. (**B**) The slower migrating from of Pto was suppressed *in trans* by prf^K1128A^ and pto^D164N^, but not by pto^S189A^ pto^T199A^ and pto^S189A/T199A^. Pto-5Myc, Pto-FLAG, pto mutant-FLAG, AvrPto, Prf-3HA, prf^K1128A^-3HA constructs were transiently expressed in wild-type *N. benthamiana* as indicated. Prf-3HA and prf^K1128A^-3HA were immunoprecipitated using anti-HA antibodies. The relative abundance of slow- and fast-migrating forms of Pto-5Myc after AvrPto recognition was quantified two days post infiltration, from anti-Myc immunoblots using Quantity One, Bio-Rad (adjusted volume = [CNT*mm2] data counts/mm^2^).

Prf forms oligomers through its novel N-term domain, bringing Pto monomers into proximity. This suggests the potential for Pto transphosphorylation [13]. To test this, we devised an assay for transphosphorylation of Pto within the Prf complex in the presence of AvrPto or avrPtoB^F479A^. The E3 ligase active AvrPtoB was not used, as it results in degradation of the kinase-dead pto^D164N^ [33]. Wild-type Pto was expressed as a fusion with five Myc epitopes (5Myc), whereas the Pto mutants described above were co-expressed as fusions with the FLAG tag, allowing differential detection of Pto or its mutant forms using appropriate antibodies. Both forms were recovered from the complex by immunoprecipitation of Prf-3HA or prf^K1128^-HA, and analysed by immunoblotting. Use of anti-Myc (to detect wild-type Pto) detected both fast and slow migrating forms in the presence of Pto-FLAG and Prf-HA, but the slow form was again suppressed by the presence of prf^K1128^-HA (Figure 3A and B). Importantly, the slow form was unaffected by the presence of the kinase-active pto^S198A^, pto^T199A^, or pto^S198A/T199A^ mutants, but was severely curtailed by kinase-dead pto^D164N^ (Figure 3 and Figure S8). These data suggest that phosphorylation of Pto within the Prf complex leading to the slow migrating form is a transphosphorylation event. Transphosphorylation between Pto molecules was previously observed in *E. coli* [22] in the absence of Prf, but in this study *in planta*, transphosphorylation required a functional Prf to induce proximity.

### Pto kinase activity is dispensable after multiple phosphorylation of the activation segment peptide

**Figure 4:**
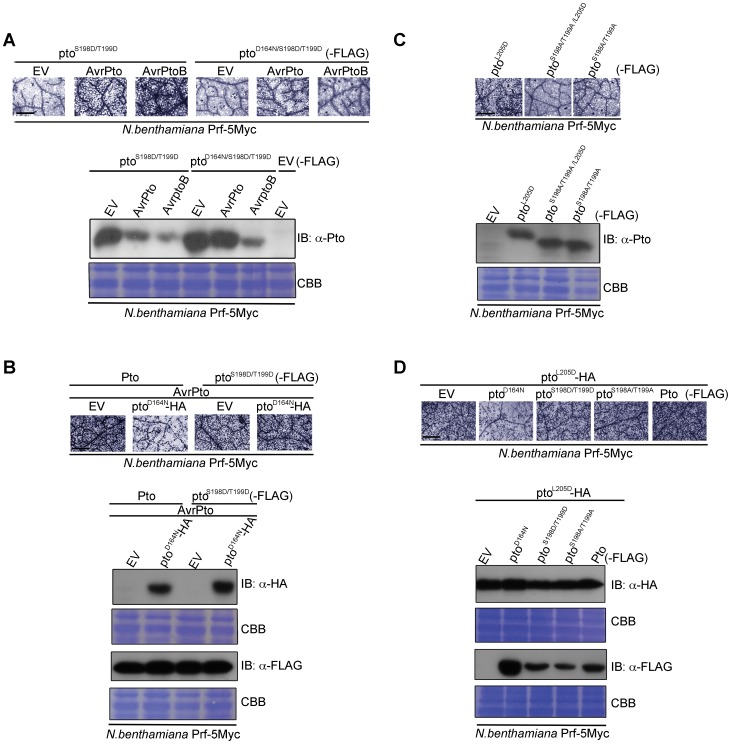
Transphosphorylation is required for signalling. (**A**) The phospho-mimic mutant pto^S198D/T199D^ induced cell death after AvrPto and AvrPtoB recognition. pto^S198D/T199D^ -FLAG, pto^D164N/S198D/T199D^-FLAG, AvrPto, and AvrPtoB constructs were transiently expressed in Pro_Prf_:*Prf-5Myc N. benthamiana* as indicated. Cell death was visualized with trypan blue staining two days post infiltration. Relative accumulation of pto^S198D/T199D^ and pto^D164N/S198D/T199D^ was detected with immunoblot (IB) with aντι-Pto antibody. Coomassie Brilliant Blue (CBB) staining of the IB membrane verified equal protein loading. (**B**) AvrPto-induced signalling by the phospho-mimic mutant pto^S198D/T199D^ is not suppressed *in trans* by pto^D164N^. Pto-FLAG, pto^S198D/T199D^-FLAG, pto^D164N^-HA and AvrPto constructs were transiently expressed in Pro_Prf_:*Prf-5Myc N. benthamiana* as indicated. Cell death was visualized as in A and relative accumulation of proteins was detected with IB with the indicated antibodies. CBB staining of the IB membranes verified equal protein loading. (**C**) Phosphorylation of the kinase-inactive, constitutive gain-of-function (CGF) mutant pto^L205D^ at Ser-198 and Thr-199 is required for its hypersensitive cell death response inducing ability. pto^L205D^-FLAG, pto^S198A/T199A/L205D^-FLAG and pto^S198A/T199A^-FLAG constructs were transiently expressed in Pro_Prf_:*Prf-5Myc N. benthamiana* as indicated. Cell death was visualized as in A and relative accumulation of proteins was detected with IB with anti-Pto antibody. CBB staining of the IB membranes verified equal protein loading. (**D**) Signalling by the kinase-inactive, CGF mutant pto^L205D^ mutant is suppressed *in trans* by pto^D164N^. pto^L205D^-HA, pto^D164N^-FLAG, pto^S198D/T199D^-FLAG, pto^S198A/T199A^-FLAG and Pto-FLAG constructs were transiently expressed in Pro_Prf_:*Prf-5Myc N. benthamiana* as indicated. Cell death was visualized as in A and relative accumulation of proteins was detected with IB with the indicated antibodies. CBB staining of the IB membranes verified equal protein loading. For all images, the bar indicates 0.5 mm. Each row of trypan blue staining is derived from a single leaf, within which relative amounts of cell death were comparable, and representative of six replicates.

We next tested whether double phosphorylation on Ser-198 and Thr-199 is sufficient for activation of Pto, as are P+1 loop CGF mutants. To do this we substituted both residues for Asp (pto^S198D/T199D^), which mimics the negative charge of phosphorylation. Expression of pto^S198D/T199D^ did not induce effector-independent HR, but the mutant was able to respond to AvrPto and AvrPtoB (Figure 4A and Figure S9A) in contrast to the pto^S198A/T199A^ variant described earlier (Figures 2A). Further introduction of the kinase-dead mutation (pto^D164N/S198D/T199D^) weakened but did not prevent recognition of AvrPto (Figure 4A and Figure S9A), in contrast to pto^D164N^ (Figure 2A). This demonstrates that Pto kinase activity is required for phosphorylation of Ser-198 and Thr-199 during AvrPto recognition, but is dispensable thereafter. Conversely, AvrPtoB was not recognised by pto^D164N/S198D/T199D^, consistent with its ability to degrade kinase inactive forms of Pto [33].

### Transphosphorylation and P+1 loop disruption are required for downstream signalling

To test the requirement for transphosphorylation in activation, we co-expressed kinase-inactive pto^D164N^ with Pto or pto^S198D/T199D^ in the presence of AvrPto. The inactive kinase suppressed signalling by wild-type Pto (Figure 4B, Figure S9B and Figure S10A), but not by the phosphomimic form pto^S198D/T199D^ (Figure 4B, Figure S9B). Thus, the kinase mutant suppressed transphosphorylation of Pto, but this effect was negated in the kinase active phosphomimic mutant. These data further show that Ser-198 and Thr-199 are the major residues in Pto that require transphosphorylation for Prf activation and downstream signalling. Lastly, substitution of Ser-198 and Thr-199 for non-phosphorylable Ala in the kinase-inactive CGF mutant pto^L205D^ (pto^S198A/T199A/L205D^) abrogated its HR-inducing ability and resulted in a marked band shift of pto^S198A/T199A/L205D^ to a fast-migrating, not phosphorylated form, in comparison to pto^L205D^ (Figure 4C and Figure S9C), suggesting that this kinase-inactive mutant must be phosphorylated *in trans*, perhaps in this system by a *N. benthamiana* Pto homolog. To test this, pto^L205D^ was co-expressed with pto^D164N^, Pto, or the substitution mutants pto^S198D/T199D^ and pto^S198A/T199A^. pto^D164N^ suppressed the pto^L205D^ CGF HR (Figure 4D, Figure S9D and Figure S10B), but the other molecules, which possess kinase activity, did not (Figure 4D and Figure S9D). Taken together, the data show that both P+1 loop disruption (through effector interaction or CGF mutation) and auto and trans phosphorylation are necessary for Pto activation.

## Discussion

We show here that activation of the Prf complex is associated with double phosphorylation of Pto kinase within its activation segment. The dual phosphorylation was seen in each signalling-active event, after effector activation or when Pto was complexed to a CGF form of Prf, and required an intact P-loop within the Prf NB subdomain. This shows that Prf is an active participant in the activation process, consistent with previous findings [23], although the role of Prf in downstream signalling is not well understood. Phosphorylation resulted in a marked band shift of Pto on SDS-PAGE to a slower migrating form. This form was never seen in the absence of tomato Prf and was detectable only when Pto was copurified with the Prf complex and further isolated with long SDS-PAGE. Therefore, in transient expression experiments where Pto is overexpressed relative to Prf, the majority of Pto within the cell is not trans-phosphorylated. Dual phosphorylation was essential for activation of Pto, could be mimicked by replacement of the phosphoresidues with Aspartate, and was destroyed by Alanine replacement. Together, our data demonstrate a strict requirement for Pto kinase activity after effector interaction. Despite this, phosphorylation was not sufficient for activation and required additional disruption of the P+1 loop.

**Figure 5:**
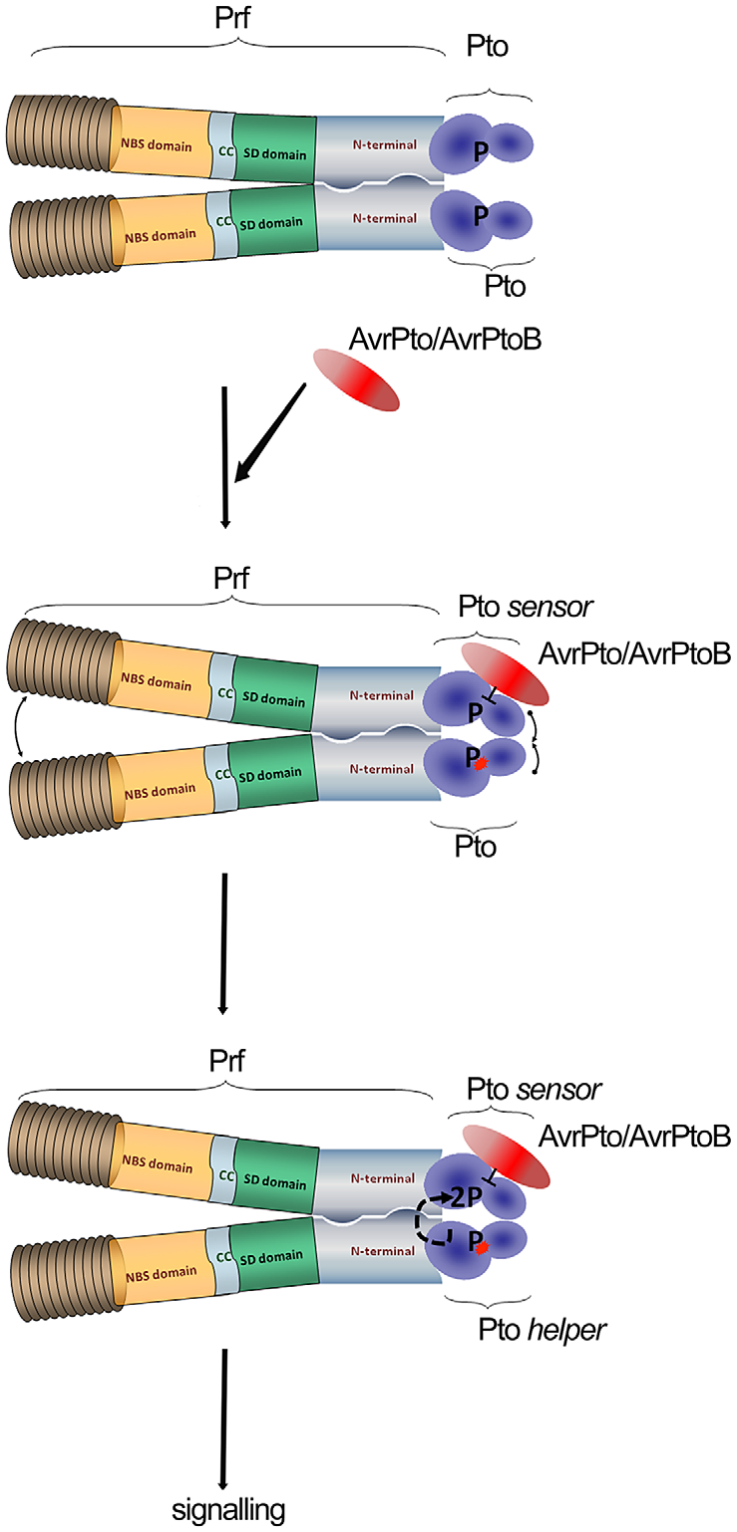
Model of sensor and helper kinases. Dimerization of the NB-LRR protein Prf bring into proximity two molecules of Pto kinase (sensor and helper). Binding of AvrPto/AvrPtoB to previously phosphorylated Pto-sensor molecule disrupts the P+1 loop and hence negative regulation imposed by Prf. Derepression of the P+1 loop activates a second Pto-helper molecule in the Prf complex that transphosphorylates the first sensor-kinase, leading to complex activation.

The role of Pto kinase activity in the function of the Prf complex has previously been obscure. We showed previously that Pto kinase activity was dispensable for binding both AvrPto and AvrPtoB [19], [20]. This interpretation was challenged by Xing et al (2007) who found that Pto in complex with AvrPto was phosphorylated on Thr-199. In their model, Thr-199 is required for effector interaction, but the kinase mutant pto^D164N^ binds both AvrPto and AvrPtoB, and the substitution mutant pto^T199A^ still recognized both effectors *in vivo* (Figure 2A). Although Pto kinase activity is dispensable for effector binding, it is clearly required for effector-mediated complex activation [19]. Interestingly, the kinase-inactive pto^D164N^ did not appear to be transphosphorylated or able to signal in most of our assays. In contrast, we showed here (Figure S7) and previously [33] that pto^D164N^ is able to initiate weak signalling after avrPtoB^F479A^ recognition suggesting that the requirement for autophosphorylation can be eventually bypassed by transphosphorylation. Nevertheless, is important to emphasize that the signalling mediated by the inactive kinase is weak and delayed (Figure S7). A model where initial autophosphorylation is not essential for effector interaction, but is a prerequisite for fast and efficient disruption of the P+1 loop could explain these discrepancies. Consistent with this model, the constitutively active P+1 loop mutant pto^L205D^ did not require kinase activity for autophosphorylation but needs to be transphosphorylated by a second kinase-active Pto for induction of cell death. Thus, co-expression of the kinase-inactive pto^D164N^ with pto^L205D^ inhibited constitutive signalling, whereas pto^D164N^ did not impair signalling by the phosphomimic pto^S198D/T199D^ mutant. Importantly, pto^S198D/T199D^ did not have a CGF phenotype suggesting that phosphorylation alone is not sufficient to activate the complex. Despite the need of validation of our model in tomato plants with bacterial-derived effectors, our data support a model in which Pto transphosphorylation after effector interaction is an essential step in activation of the recognition complex, but is not sufficient for complex activation which requires further disruption of the kinase P+1 loop (Figure 5 and Table S3).

Our data provides linked explanations for two phenomena: Why Prf exists in a multimeric complex, and secondly, how the kinase inhibitor AvrPto activates the Prf complex in a manner dependent on Pto kinase activity. In crystal structures, AvrPto binds to the Pto catalytic cleft occluding the active site, and inhibits Pto kinase activity *in vitro*. Likewise, AvrPto inhibits the kinase activities of many PRRs. How then can a kinase inhibitor act as an activator of the Pto/Prf complex? We proposed the following model derived in large part from the current data set. Binding of AvrPto to Prf-associated, previously autophosphorylated sensor Pto disrupts the P+1 loop and hence the negative regulation imposed by Prf [23]. Derepression of the P+1 loop activates a second helper Pto molecule in the Prf complex, either directly or indirectly, mediated through the Prf NB moiety. The second kinase molecule transphosphorylates the first, leading to full activation of the complex. It is tempting to speculate that similar transphosphorylation events within the activated complex lead to phosphorylation of downstream targets of Pto.

This mechanism could not work unless the Prf complex was multimeric. In a monomeric complex, the outcome of effector-Pto interaction would be kinase inhibition, as has been shown for the PRRs. In contrast, our results separate alternate Pto moieties as either sensor or helper kinases depending on which perceives the effector molecule. Such a mechanism is most likely to be successful at early stages of infection when the molecules of Pto/Prf will always outnumber the effector molecules. In this way, Pto acts as the bait in a molecular trap for effectors, which target protein kinase domains. This idea is particularly compelling because of the high similarity between Pto and the kinase domains of most plant receptor kinases, notably CERK1 [10], many of which are likely to be PRRs. We speculate that the Pto complex evolved subsequent to evolution of pathogen effectors that target PRR domains. Indeed, it is tempting to speculate further that Pto itself was derived from duplication of a genetic fragment encoding a targeted PRR kinase domain, and that the novel Prf N-term domain evolved to exploit the kinase-effector interaction which was evolved previously by the pathogen. All pathogens need to suppress PRR kinases, so in this context, the Pto/Prf complex is an ingenious molecular trap for kinase-tropic effectors.

Another example of a protein kinase targeted by an effector protein and interacting with a NB-LRR protein is the *Arabidopsis* PBS1. The *Pseudomonas syringae* effector protein AvrPphB is a cysteine protease that targets PBS1 for cleavage. The NB-LRR protein RPS5 monitors the integrity of PBS1 and is activated upon PBS1 cleavage by AvrPphB [14], [34], [35]. Similarly to Prf, RPS5 forms dimers or oligomers [14]; but in contrast to the Pto/Prf complex no need for transphosphorylation has been demonstrated within the PBS1/RPS5 complex. Initially it was shown that the PBS1 kinase activity was required for RPS5 activation [34], [36] but more recent results indicated that the kinase activity of PBS1 is dispensable for signalling [37]. The authors proposed a model where the NB-LRR protein is activated by conformational changes of the guard (sensor) protein caused by the effector protein, without the need of kinase activity [37]. Our data do not quite fit this model because Pto is clearly functional with a requirement for kinase activity documented here, and Prf plays an intimate co-regulatory role with Pto. In our model the NB-LRR protein is activated in the presence of the effector by simultaneously triggered conformational changes and transphosphorylation of the guarded (sensor) kinase. Thus Pto acts a sophisticated bait for the effector, based on its kinase activity and its highly relatedness to the kinase domains of PRRs.

## Materials and Methods

### Transient gene expression, transgenic lines and cell death assay

Growth and transient expression conditions for *N. benthamiana* were as described [20] using the *A. tumefaciens* strain C58C1. Transgenic *N. benthamiana* lines used were wt11c containing Pro_Prf_:*Prf-5Myc* [24] and 38-12 containing 35S:*Pto* [38]. Qualitative cell death assays were performed by boiling leaves in lactophenol trypan blue solution including 60% ethanol and clearing with chloral hydrate. Cell death stains dark blue in this assay. Cell death was estimated as Relative HR Index using ImageJ, as the darkly stained area in a total leaf area of 0.5 mm^2^ (vascular tissue was omitted from the calculations). The cell death was estimated using images of cell death from three independent experiments using two leaves in each experiment. All pictures were taken two days post infiltration.

### Co-immunoprecipitation assays

For the analysis of protein accumulation from *N. benthamiana*, leaves frozen in liquid nitrogen were added to extraction buffer, 150 mM Tris-HCl (pH 7.5), 150 mM NaCl, 5 mM EDTA, 5% glycerol (v/v), 10 mM dithiothreitol (DTT), 2% polyvinylpolypyrrolidone (PVPP), 1% plant protease inhibitor cocktail (Sigma), and 0.5 mM PMSF and homogenized with a Polytron. Protein extracts were centrifuged at 20,000 g for 20 min at 4°C. Supernatants were subjected to filtration through a 0.45 µm filter. Sepharose affinity matrices used were anti-FLAG M2 and anti-HA HA-7 (both Sigma). Extracts were mixed with affinity matrices as indicated for two hours at 4°C, with gentle rotation, in batch format. Affinity matrices were washed three times with an excess of extraction buffer. Proteins were stripped from the bead fraction by boiling in SDS loading buffer. Elution from anti-FLAG beads was performed by incubation with extraction buffer containing 200 µg/mL FLAG peptide (Sigma) for 10 min at 25°C with gentle mixing. To concentrate elutions, Strataclean (Stratagene) beads were added to bind proteins, and then pelleted by centrifugation subsequently the proteins were stripped from the beads by boiling in 1× SDS-PAGE loading buffer.

### Kinase assays

In vitro kinase assays were performed as described [39] with slight modifications. Briefly, the kinase reaction mixture contained 50 mM Tris-HCl (pH 7.5), 10 mM MgCl_2_, 1 mM MnCl_2_, 1 mM DTT, 20 µM ATP, 183 kBq of γ[^32^P]-ATP (PerkinElmer Life Sciences) in a total volume of 30 µl. In Pto transphosphorylation assays, Pto-FLAG, pto^D164N^-FLAG, pto^S198A^-FLAG, pto^S199A^-FLAG and pto^S198A/T199A^-FLAG were transiently expressed in *N. benthamiana* and immunoprecipitated with anti-FLAG beads as described above. pti1^K96N^-His and GST-AvrPtoB were expressed and purified from *Escherichia coli* as previously [33]. 5 µg of pti1^K96N^-His and 2 µg of GST-AvrPtoB were included in the kinase reaction mixture. All reactions were initiated by addition of the kinase mixture, incubated at 30°C for 20 min, and terminated by addition of SDS-polyacrylamide gel electrophoresis (SDS-PAGE) loading buffer and boiling for 10 min. Under these assay conditions, incorporation of radiolabel was found to be linear with time, the substrate and the enzyme concentration used. At the end of each assay, samples were loaded onto SDS-PAGE. Post electrophoresis, proteins were transferred onto polyvinylidene difluoride membranes and stained with Coomassie Brilliant Blue R-250. Subsequently, the membranes were subjected to autoradiography using a FUJI Film FLA5000 PhosphorImager (Fuji, Tokyo, Japan). Relative autophosphorylation and transphosphorylation kinase activity was calculated as the ratio between incorporated radioactivity (PhosphorImager signal) and the amount of immunoprecipitated protein estimated using ImageJ based on Coomassie staining of the membranes and expressed as a percentage of Pto-FLAG relative autophosphorylation or transphosphorylation kinase activity.

### Identification of proteins by mass spectrometry

Co-immunoprecipitated protein complexes were separated by SDS-PAGE and gel slices were excised. Proteins were reduced with DTT and alkylated with iodoacetamide before in-gel trypsin (Promega) digestion overnight at 37°C. After digestion, the supernatant was moved to a clean tube and the gel pieces washed sequentially with 50% and 100% acetonitrile and the washes pooled with the supernatant. Volume and the organic content of the peptide solution were reduced by lypholisation and the peptides stored at −20°C until use. Peptides were dissolved in 0.5% formic acid immediately before analysis by LC MS/MS.

LC-MS/MS analysis was performed using a LTQ-Orbitrap XL mass spectrometer (Thermo Scientific) and a nanoflow-HPLC system (Surveyor, Thermo Scientific). Peptides were applied to a precolumn (C18 pepmap100, LC Packings) connected to a self-packed C18 10-cm analytical column (BioBasic resin, Thermo Scientific. Picotip 75 µm id, 15 µm tip, New Objective). Peptides were eluted by a gradient of 2 to 50% acetonitrile in 0.1% formic acid over 50 min at a flow rate of approximately 250 nL min-1. Data-dependent acquisition of MS/MS consisted of selection of the five most abundant ions in each cycle: MS mass-to-charge ratio (*m/z*) 300 to 2000, minimum signal 1000, collision energy 35, 2 repeat hits, 60 sec exclusion. MS3 were triggered if the neutral loss of phosphoric acid (49 *m/z* for 2+ parent ions) was detected in the three most abundant ions on the preceding MS2. Collision energy for MS3 was 35. In all cases the mass spectrometer was operated in positive ion mode with a nano-spray source and a capillary temperature of 200°C, no sheath gas was employed and the source and focusing voltages were optimised for the transmission of angiotensin.

Peak lists (as .dat files) were prepared from raw data using extract_msn in BioWorks 3.3 (Thermo Electron Corp.) and collated using merge.pl (Matrix Science). The data generation parameters were: MW range 300.00–3500.00, threshold absolute 1000, group scan 10, minimum group 0, minimum ion cont 10, charge state auto (ZSA processing; default values) MS level auto. Peak lists were searched against SPtrEMBL, (containing 8385695 sequences) Taxonomy was restricted to Solaneae (7247 sequences) with the following variable modifications were allowed; oxidized methionine, phosphorylation on serine and threonine. Carbamidomethyl was specified as a fixed modification on cysteine residues. Precursor mass tolerance was 5 ppm, fragment tolerance 0.5 Da mass values were monoisotopic and two missed tryptic cleavages were allowed. Subsequently for proteins identified with >95% probability an ‘error tolerant’ search was preformed in Mascot with relaxed criteria for modifications and enzyme cleavage. Scaffold (version Scaffold_2_03_01, Proteome Software Inc.) was used to validate MS/MS based peptide and protein identifications. Peptide identifications were accepted if they could be established at greater than 95.0% probability as specified by the Peptide Prophet algorithm [40]. Protein identifications were accepted if they could be established at greater than 95.0% probability and contained at least 2 identified peptides. Protein probabilities were assigned by the Protein Prophet algorithm [41].

### Assignment of phosphorylation sites and spectrum counting

In addition to the minimum PeptideProphet score provided by Scaffold, we manually evaluated the fragmentation spectra of all phosphorylated peptides of Pto to ensure that good b and y ion coverage was observed and that neutral loss of the phosphate (common in ion trap CID) supported the assigned phosphorylated residue. We used our PhosCalc algorithum to assist this interpretation (MacLean et al 2008). The peptides covering the activation loop (KGTELDQTHLSTVVK) were observed with and without a tryptic miscleavage of the N-terminal K, and single and double phosphorylation events were observed on both tryptic fragments. Furthermore, the single miscleaved peptide was observed in both 2+ and 3+ ionisation states, while most other peptides were predominantly 2+, thus providing abundant evidence of mass shifts and fragmentation patterns. This peptide contains four possible phosphorylation sites. We observed spectra, which supported single phosphorylation at T190, S198 or T199. Site S198 was most strongly supported and frequently observed. We also observed a doubly phosphorylated form of KGTELDQTHLSTVVK, again with and without the N-terminal miscleavage. Manual examination of the spectra supported phosphorylation at S198 and T199 in most cases, occasional spectra supported phosphorylation at T190 and either S198 or T199. It is important to note that even in cases where the exact site of modification remains ambiguous this does not detract from the double phosphorylation of the peptide as a whole, due to the clear difference in parent ion masses and overall fragmentation. Representative fragmentation spectra for the peptide (KGTELDQTHLSTVVK) are shown in Supplementary Figures S2 and S3. Spectrum counts were summed over both cleavage forms and scored as unmodified, single or double phosphorylation, all counted spectra were at the 95% peptide prophet threshold provided by Scaffold.

### Accession numbers

Sequence data from this article can be found in the GenBank data library under the following accession numbers: Prf (U65391), Pto (DQ019170), avrPto (L20425), avrPtoB (Q8RSY1).

## Supporting Information

Figure S1 **Inhibition of Pto/Prf–mediated signalling** ***in trans*** **.** (**A**) The Pto/Prf ligand-independent hypersensitive cell death response (HR) in *N. benthamiana* is compromised by co-expression of the loss-of-function mutant prf^K1128A^. The indicated Prf-5Myc, prf^K1128A^-3HA, Cp-GFP and Rx-HA constructs were transiently expressed in stable transgenic 35S:*Pto* (no tag) *N. benthamiana* plants. As a control for specificity, prf^K1128A^ was co-expressed with CP-GFP and Rx-HA. The picture was taken at three days post infiltration. Protein expression was confirmed by immunoblots (IB) with the antibodies indicated on the right. Coomassie Brilliant Blue (CBB) staining of the IB membrane verified equal protein loading. The experiment was repeated several times and typical results are shown. (**B**) AvrPto-induced hypersensitive cell death response is compromised by co-expression of the loss-of-function mutant prf^K1128A^. prf^K1128A^-3HA, Pto-Flag, pto^D164N^-FLAG and AvrPto constructs were transiently expressed in stable transgenic Pro_Prf_:*Prf-5Myc N. benthamiana* leaves as indicated and the tissue was stained with trypan blue 2 days post infiltration. The bar indicates 0.5 mm. Dead cells stain dark blue in this assay.
(TIF)

Figure S2 **MS spectra of the Pto peptides 188–202 (GTELDQTHLSTVVK).** (**A**) Spectra supporting single phosphorylation on S198 (Mascot score 85.96) (**B**) Spectra supporting single phosphorylation on T199 (Mascot score 49.23). (**C**) Spectra supporting double phosphorylation on S199 and T199 (Mascot score 49.33).
(TIF)

Figure S3 **MS spectra of the Pto peptides 187–202 (KGTELDQTHLSTVVK).** (**A**) Spectra supporting single phosphorylation on S198 (Mascot score 84.08) (**B**) Spectra supporting single phosphorylation on T199 (Mascot score 54.7). (**C**) Spectra supporting double phosphorylation on S199 and T199 (Mascot score 62.61).
(TIF)

Figure S4 **Phosphorylation on Pto residues S198 and T199 is required for induction of cell death.** (**A,B,C,D**) Relative Hypersensitive Response (HR) index was estimated from trypan blue staining of cell death in *N. benthamiana* leaves based on three independent experiments. Cell death stains dark blue in this qualitative assay and was estimated using ImageJ. The proteins were transiently expressed as indicated and pictures were taken two days post infiltration. Representative pictures are in Figure 2A. Each graph is derived from three leaves, within which relative amounts of cell death were comparable. Error bars are standard deviation.
(TIF)

Figure S5 **Inhibition of Prf CGF HR** ***in trans*** **.** (**A**) The prf^D1416V^ constitutive gain-of-function (CGF) phenotype in *N. benthamiana* is compromised by co-expression of the loss-of-function mutant prf^K1128A^. The indicated prf^K1128A^-3HA, prf^D1416V^-5Myc, Cp-GFP and Rx-HA constructs were transiently expressed in *N. benthamiana* plants. As a control for specificity, prf^K1128A^ was co-expressed with CP-GFP and Rx-HA. The picture was taken at three days post infiltration. Protein expression was confirmed by immunoblots (IB) with the antibodies indicated on the right. Coomassie Brilliant Blue (CBB) staining of the IB membrane verified equal protein loading. The experiment was repeated several times and typical results are shown. (**B**) The K1128A and D1416V mutations do not affect the ability of Prf variants to dimerise. Transgenic 35S:Pto *N. benthamiana* leaves were transiently transformed with prf^K1128A^-5Myc alone, or in combination with Prf-3HA, prf^D1416V^-3HA, or prf^K1128A^-3HA. Infiltrated leaves were collected two days post infiltration for extraction of proteins. Tagged proteins were immunoprecipitated using anti-HA beads. Crude extract (input) and beads fractions were analysed by SDS-PAGE followed by immunoblotting with the antibodies indicated on the right. Equal protein loading was confirmed by CBB staining of the immunoblot membrane.
(TIF)

Figure S6 **Mutants of S198 and T199 are kinase active forms of Pto.** Kinase activity assays showing Pto autophosphorylation and transphosphorylation of Pti1^K96N^ and AvrPtoB. Pto-FLAG, pto^D164N^-FLAG, pto^S198A^-FLAG, pto^T199A^-FLAG, and pto^S198A/T199A^-FLAG proteins were transiently expressed in *N. benthamiana* and immunoprecipitated with anti-FLAG M2 affinity matrix. pti1^K96N^-His and GST-AvrPtoB substrates were expressed and purified from *Escherichia coli*. Coomassie Brilliant Blue (CBB) staining of the immunoblot (IB) membranes verified that equal amounts of pti1^K96N^-His and GST-AvrPtoB were added in each assay. Relative autophosphorylation and transphosphorylation kinase activity was calculated as the ratio between incorporated radioactivity and the amount of immunoprecipitated protein estimated based on CBB staining of the IB membrane and expressed as a percentage of Pto-FLAG relative autophosphorylation or transphosphorylation kinase activity.
(TIF)

Figure S7 **Double phosphorylation on S198 and T199 is necessary for signalling.** (**A**) Trypan blue staining of cell death in *N. benthamiana* leaves. Pto-FLAG, pto mutant-FLAG and avrPtoB^F479A^ constructs were transiently expressed in stable transgenic Pro_Prf_:*Prf-5Myc N. benthamiana* leaves as indicated and the tissue was stained 24 or 48 hours post infiltration. The bar indicates 0.5 mm. Dead cells stain dark blue in this qualitative assay. Each row is derived from a single leaf, within which relative amounts of cell death were comparable, and is representative of three replicates. (**B**) Relative Hypersensitive Response (HR) index. Images of trypan blue staining of cell death in *N. benthamiana* leaves from three independent experiments were used to estimate the Relative HR index. The proteins were transiently expressed as indicated and pictures were taken as indicated in A. Each graph is derived from three leaves, within which relative amounts of cell death were comparable. Error bars are standard deviation.
(TIF)

Figure S8 T**he slower migrating from of Pto was suppressed** ***in trans*** **by pto^D164N^, but not by pto^S189A/T199A^.** Prf-3HA, Pto-5Myc, Pto-FLAG, pto^D164N^-FLAG, pto^S189A/T199A^ -FLAG and avrPtoB^F479A^ constructs were transiently expressed in wild-type *N. benthamiana* as indicated. The E3 ligase mutant avrPtoB^F479A^ was used instead of wild-type AvrPtoB to avoid pto^D164N^ degradation. Prf-3HA was immunoprecipitated (IP) using anti-HA antibodies. The relative abundance of slow- and fast-migrating forms of Pto-FLAG, pto^D164N^-FLAG, pto^S189A/T199A^ -FLAG were quantified using anti-FLAG immunoblots (IB) (upper graph). Anti-Myc immunoblots (lower graph) were used for quantification of slow- and fast-migrating forms of Pto-5Myc in the presence of different Pto variants. Quantity One, Bio-Rad (adjusted volume = [CNT*mm2] data counts/mm^2^) was used for quantification of anti-FLAG and anti-Myc immunoblots. Error bars are standard deviation of relative abundance between the same samples in independent immunoblots, probed with the anti-FLAG or anti-Myc antibodies.
(TIF)

Figure S9 **Transphosphorylation is required for induction of cell death.** (**A,B,C,D**) Relative Hypersensitive Response (HR) index was estimated from trypan blue staining of cell death in *N. benthamiana* leaves based on three independent experiments. Cell death stains dark blue in this qualitative assay and was estimated using ImageJ. The proteins were transiently expressed as indicated and pictures were taken two days post infiltration. Representative pictures are in Figure 4. Each graph is derived from three leaves, within which relative amounts of cell death were comparable. Error bars are standard deviation.
(TIF)

Figure S10 **The kinase inactive pto^D164N^ compromises hypersensitive cell death response (HR)** ***in trans*** **.** (**A**). The Hypersensitive Response (HR) triggered by AvrPto recognition is compromised by kinase-inactive pto^D164N^. The indicated AvrPto, Pto-5Myc and pto^D164N^-HA constructs were transiently expressed in stable transgenic Pro_Prf_:*Prf-5Myc N. benthamiana* leaves. The picture was taken at three days post infiltration. Protein expression was confirmed by immunoblots (IB) with the antibodies indicated on the right. Coomassie Brilliant Blue (CBB) staining of the IB membrane verified equal protein loading. The experiment was repeated several times and typical results are shown. (**B**) The cell death phenotype triggered by the constitutive gain-of-function (CGF) mutant pto^L205D^ is compromised by the co-expression of kinase-inactive pto^D164N^. The indicated pto^L205D^-GFP, Pto-5Myc and pto^D164N^-HA constructs were transiently expressed in stable transgenic Pro_Prf_:*Prf-5Myc N. benthamiana* leaves. The picture was taken at three days post infiltration. Protein expression was confirmed by immunoblots with the antibodies indicated on the right. CBB staining of the IB membrane verified equal protein loading. The experiment was repeated several times and typical results are shown.
(TIF)

Table S1 **Double phosphorylation of Pto peptide 187–202 and 1888-202 upon activation of signalling.**
(DOCX)

Table S2 **Double phosphorylation of Pto peptide 187–202 and 188–202 upon activation of signalling.**
(DOCX)

Table S3 **Summary of the different complex combinations examined, and their signalling outcomes.**
(DOCX)
