# Supplementary figures and images for: The Tomato Prf Complex Is a Molecular Trap for Bacterial Effectors Based on Pto Transphosphorylation
Source: PLoS Pathog. 2013 Jan 31;9(1):e1003123. doi: 10.1371/journal.ppat.1003123 (PMC3561153; doi:10.1371/journal.ppat.1003123)

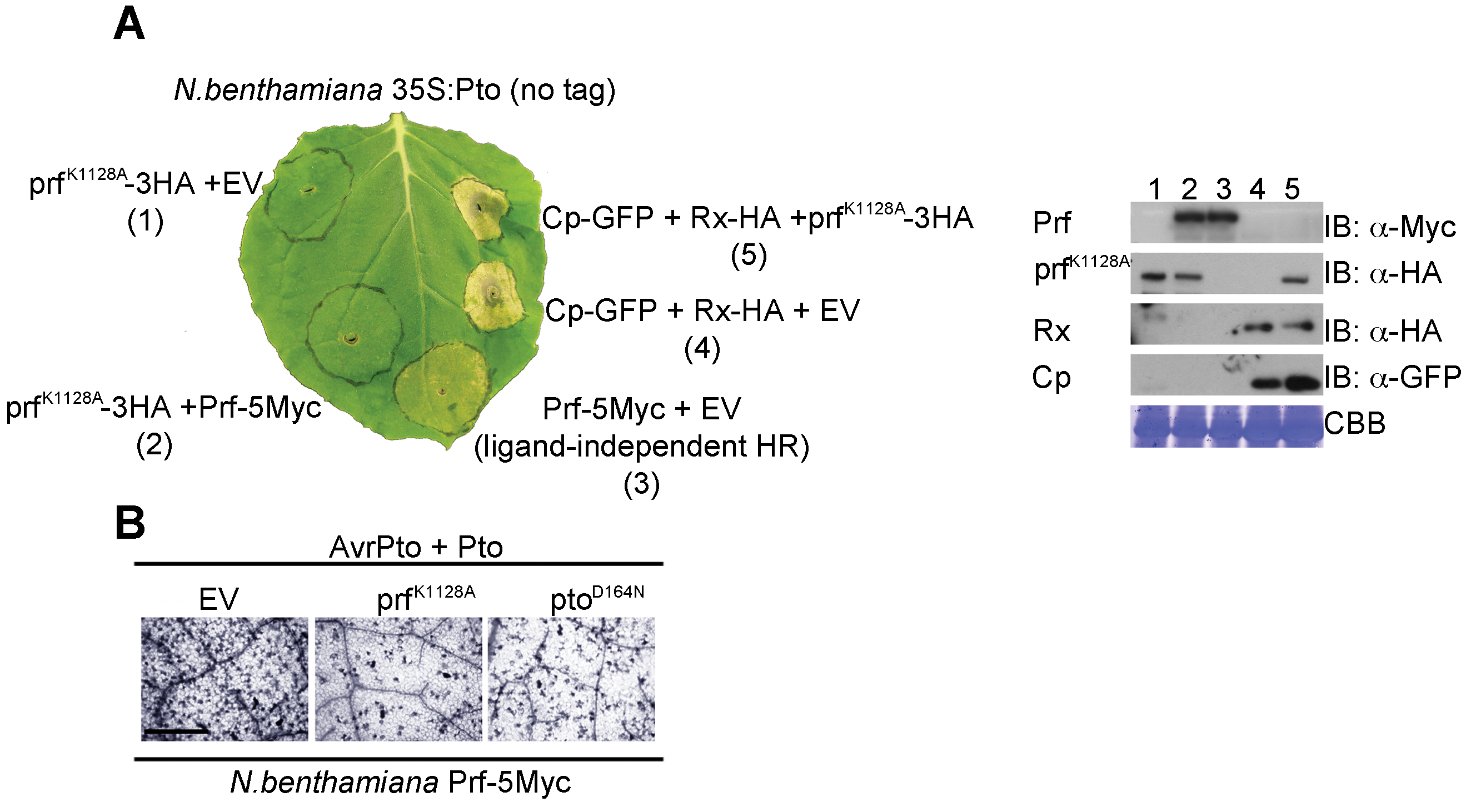

Supplement: Figure S1 — Inhibition of Pto/Prf–mediated signalling in trans . (A) The Pto/Prf ligand-independent hypersensitive cell death response (HR) in N. benthamiana is compromised by co-expression of the loss-of-function mutant prfK1128A. The indicated Prf-5Myc, prfK1128A-3HA, Cp-GFP and Rx-HA constructs were transiently expressed in stable transgenic 35S:Pto (no tag) N. benthamiana plants. As a control for specificity, prfK1128A was co-expressed with CP-GFP and Rx-HA. The picture was taken at three days post infiltration. Protein expression was confirmed by immunoblots (IB) with the antibodies indicated on the right. Coomassie Brilliant Blue (CBB) staining of the IB membrane verified equal protein loading. The experiment was repeated several times and typical results are shown. (B) AvrPto-induced hypersensitive cell death response is compromised by co-expression of the loss-of-function mutant prfK1128A. prfK1128A-3HA, Pto-Flag, ptoD164N-FLAG and AvrPto constructs were transiently expressed in stable transgenic ProPrf:Prf-5Myc N. benthamiana leaves as indicated and the tissue was stained with trypan blue 2 days post infiltration. The bar indicates 0.5 mm. Dead cells stain dark blue in this assay. (TIF) [file ppat.1003123.s001.tif]

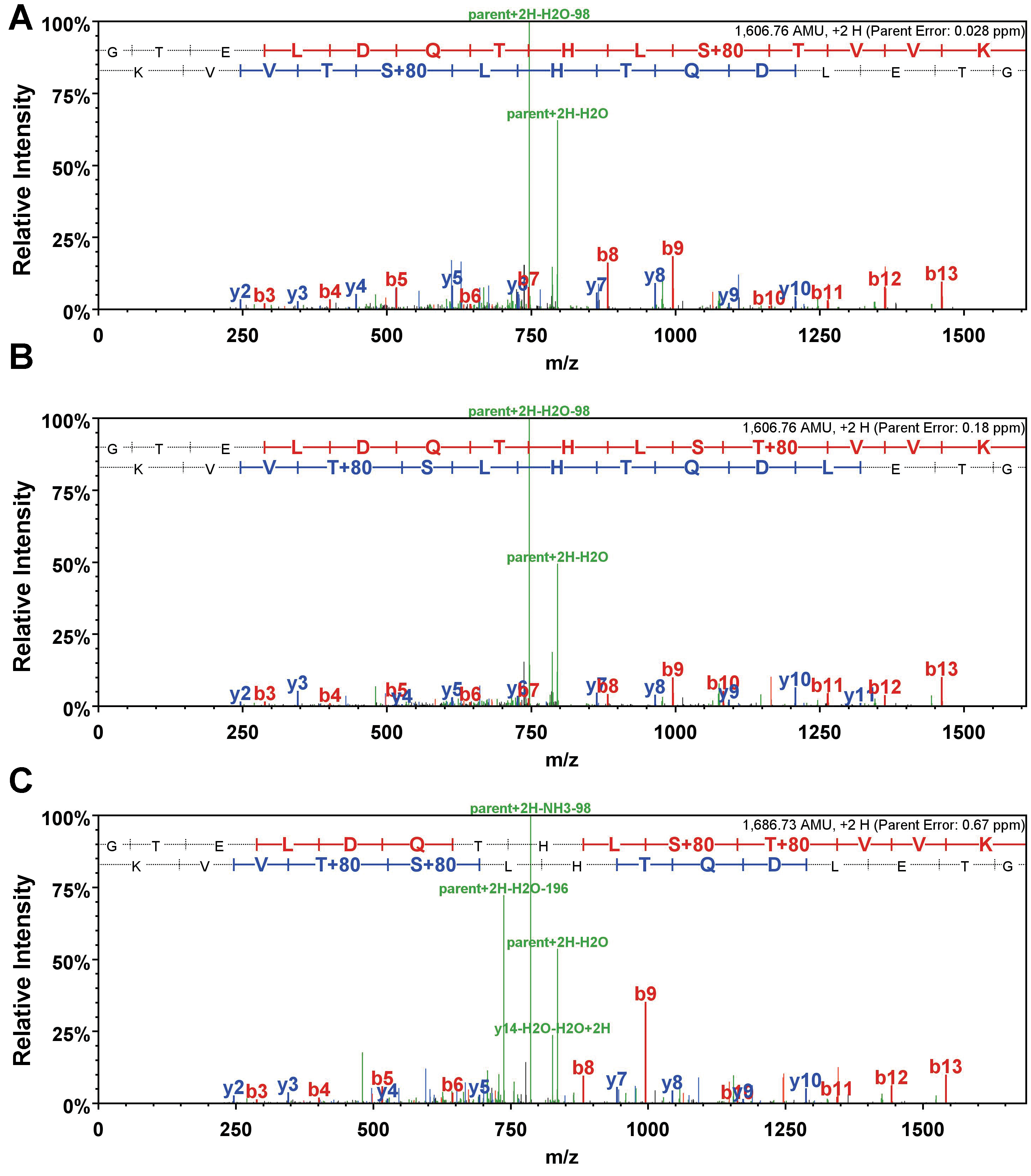

Supplement: Figure S2 — MS spectra of the Pto peptides 188–202 (GTELDQTHLSTVVK). (A) Spectra supporting single phosphorylation on S198 (Mascot score 85.96) (B) Spectra supporting single phosphorylation on T199 (Mascot score 49.23). (C) Spectra supporting double phosphorylation on S199 and T199 (Mascot score 49.33). (TIF) [file ppat.1003123.s002.tif]

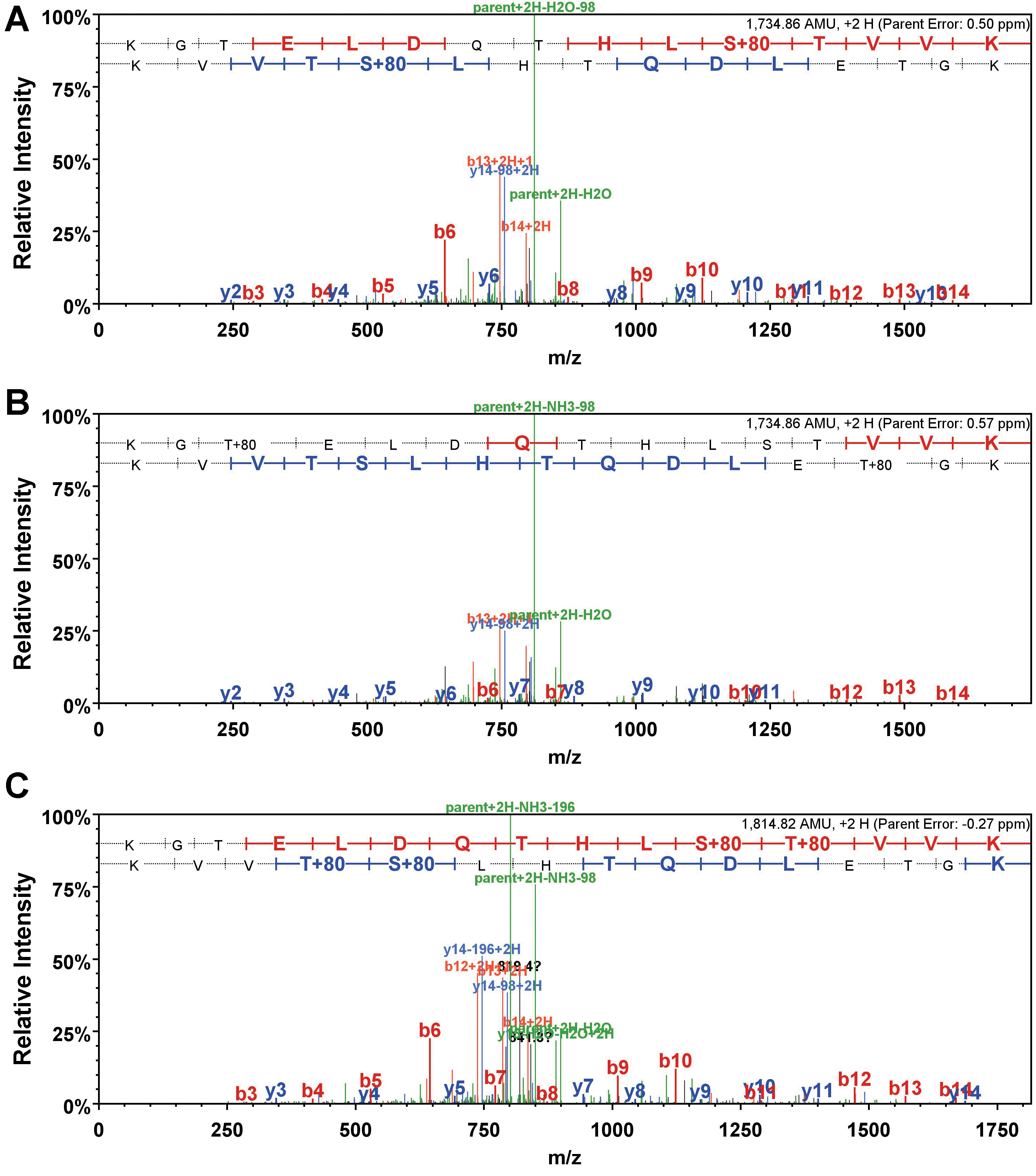

Supplement: Figure S3 — MS spectra of the Pto peptides 187–202 (KGTELDQTHLSTVVK). (A) Spectra supporting single phosphorylation on S198 (Mascot score 84.08) (B) Spectra supporting single phosphorylation on T199 (Mascot score 54.7). (C) Spectra supporting double phosphorylation on S199 and T199 (Mascot score 62.61). (TIF) [file ppat.1003123.s003.tif]

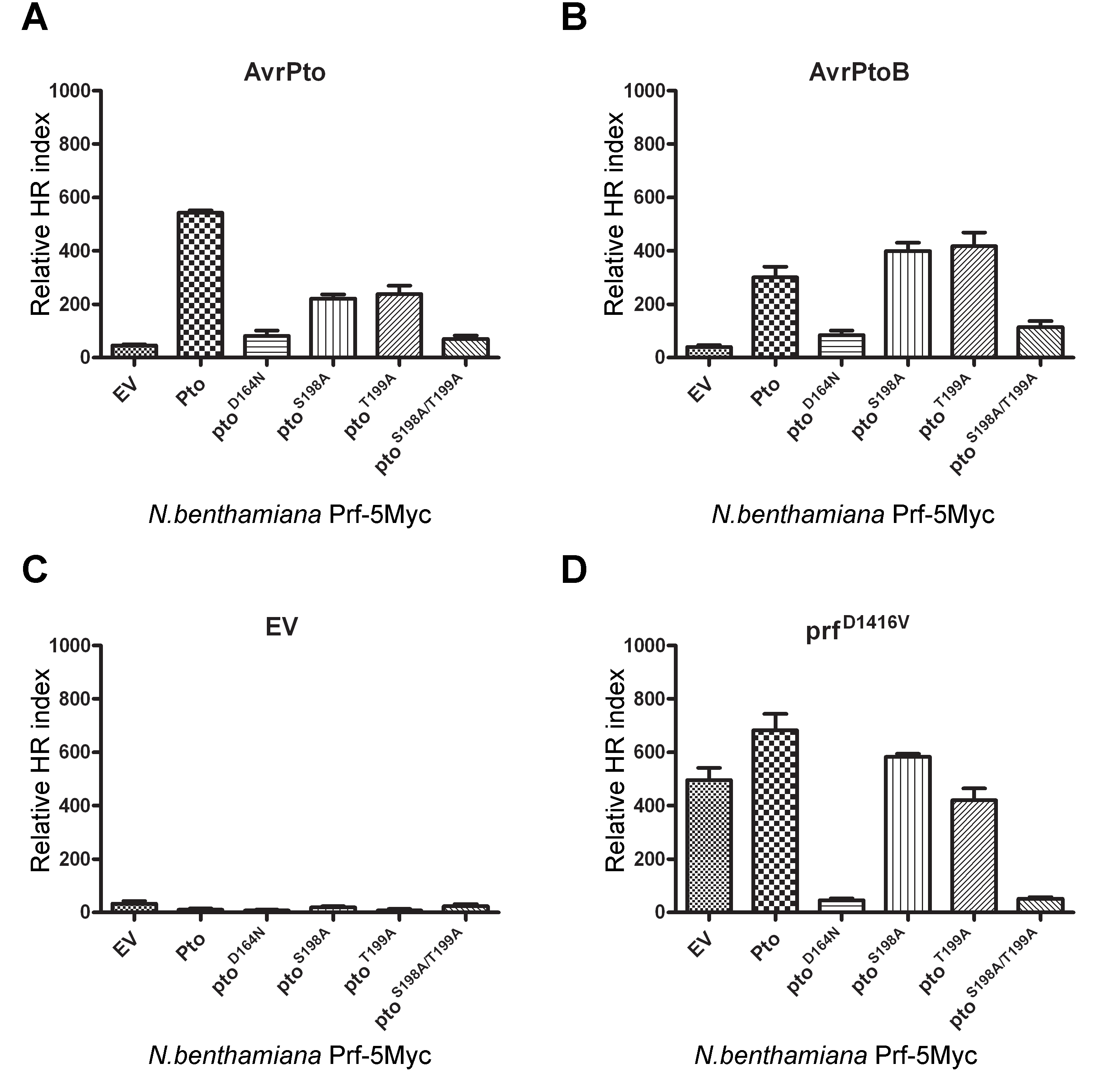

Supplement: Figure S4 — Phosphorylation on Pto residues S198 and T199 is required for induction of cell death. (A,B,C,D) Relative Hypersensitive Response (HR) index was estimated from trypan blue staining of cell death in N. benthamiana leaves based on three independent experiments. Cell death stains dark blue in this qualitative assay and was estimated using ImageJ. The proteins were transiently expressed as indicated and pictures were taken two days post infiltration. Representative pictures are in Figure 2A. Each graph is derived from three leaves, within which relative amounts of cell death were comparable. Error bars are standard deviation. (TIF) [file ppat.1003123.s004.tif]

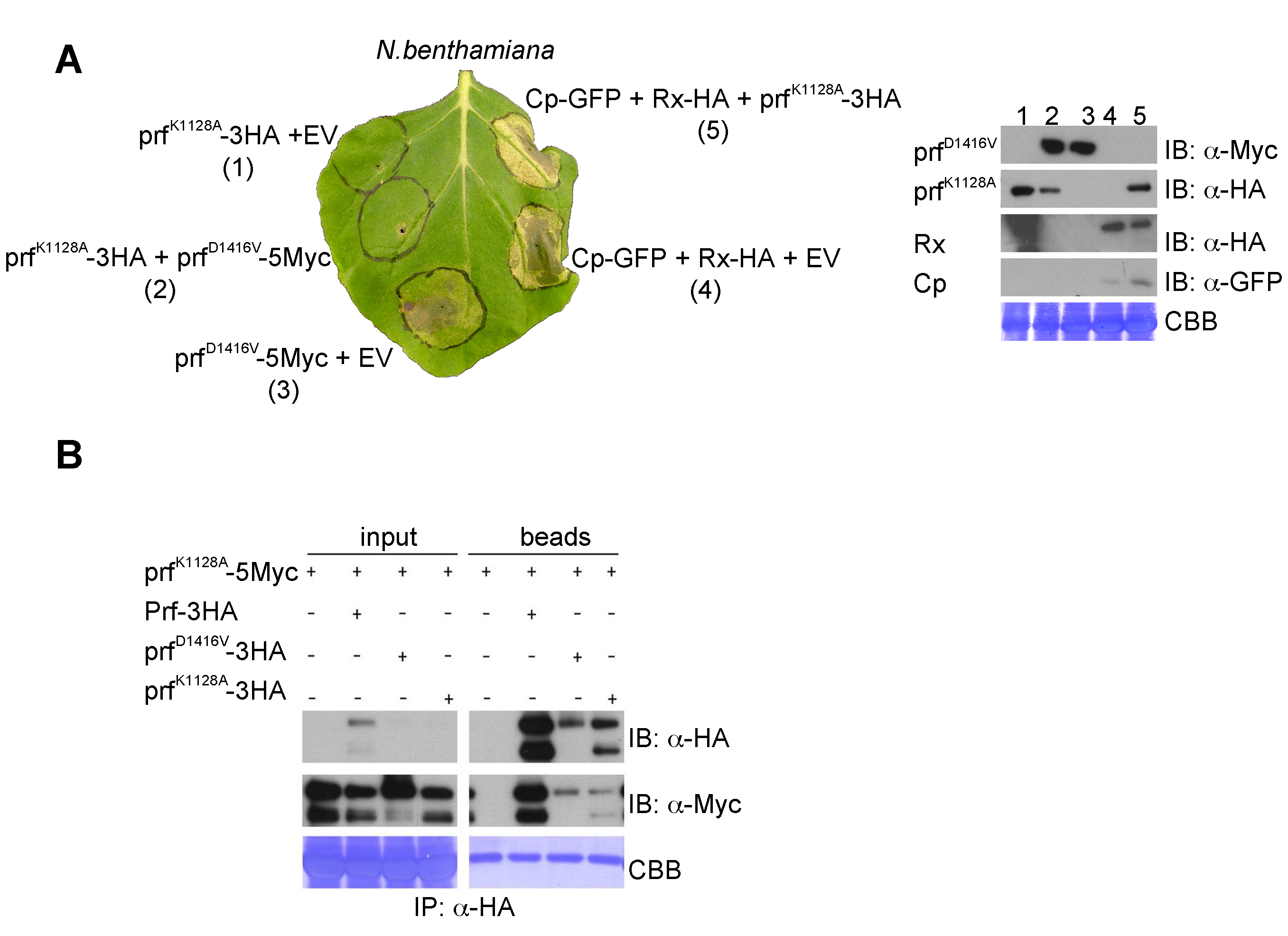

Supplement: Figure S5 — Inhibition of Prf CGF HR in trans . (A) The prfD1416V constitutive gain-of-function (CGF) phenotype in N. benthamiana is compromised by co-expression of the loss-of-function mutant prfK1128A. The indicated prfK1128A-3HA, prfD1416V-5Myc, Cp-GFP and Rx-HA constructs were transiently expressed in N. benthamiana plants. As a control for specificity, prfK1128A was co-expressed with CP-GFP and Rx-HA. The picture was taken at three days post infiltration. Protein expression was confirmed by immunoblots (IB) with the antibodies indicated on the right. Coomassie Brilliant Blue (CBB) staining of the IB membrane verified equal protein loading. The experiment was repeated several times and typical results are shown. (B) The K1128A and D1416V mutations do not affect the ability of Prf variants to dimerise. Transgenic 35S:Pto N. benthamiana leaves were transiently transformed with prfK1128A-5Myc alone, or in combination with Prf-3HA, prfD1416V-3HA, or prfK1128A-3HA. Infiltrated leaves were collected two days post infiltration for extraction of proteins. Tagged proteins were immunoprecipitated using anti-HA beads. Crude extract (input) and beads fractions were analysed by SDS-PAGE followed by immunoblotting with the antibodies indicated on the right. Equal protein loading was confirmed by CBB staining of the immunoblot membrane. (TIF) [file ppat.1003123.s005.tif]

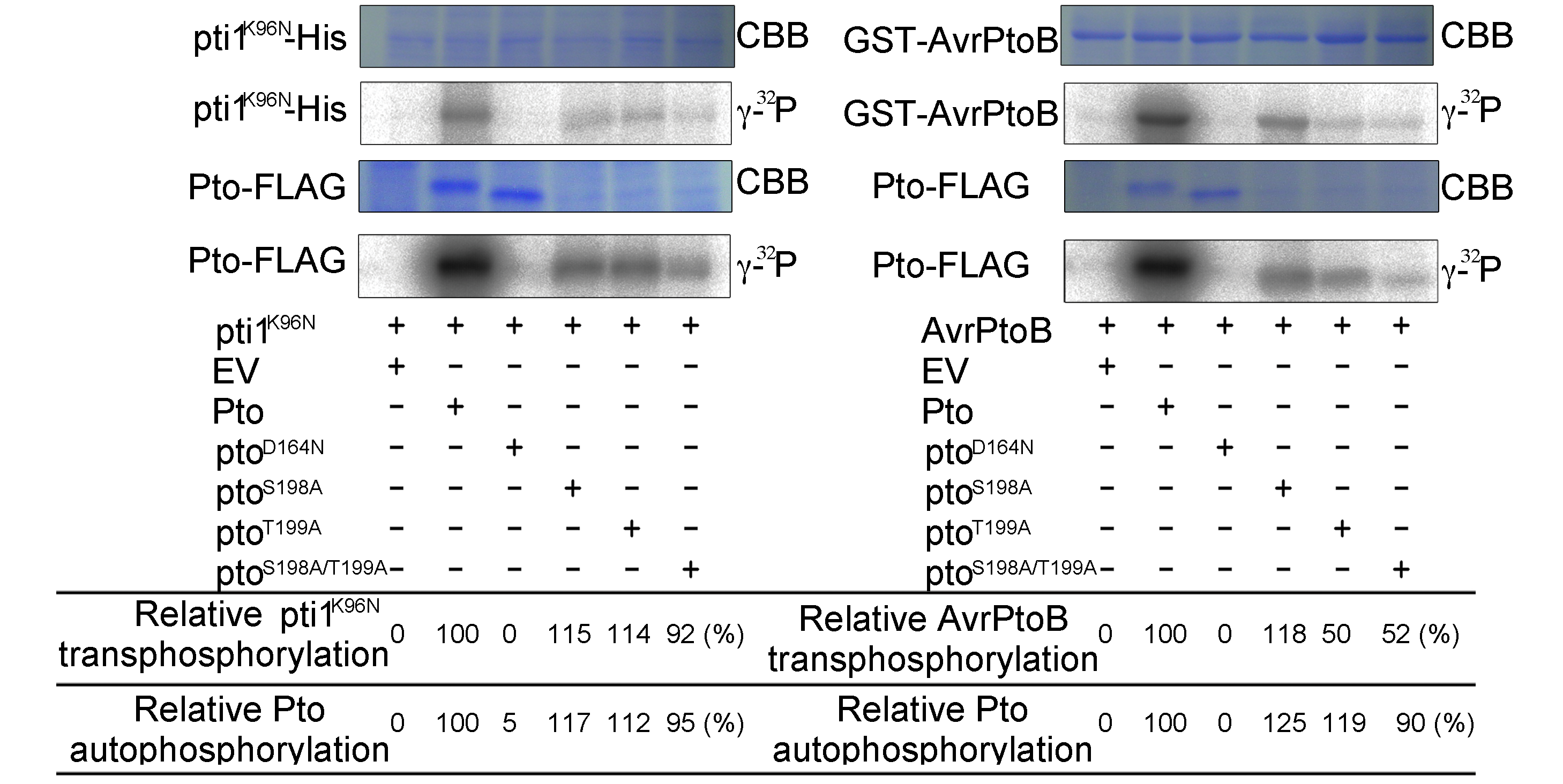

Supplement: Figure S6 — Mutants of S198 and T199 are kinase active forms of Pto. Kinase activity assays showing Pto autophosphorylation and transphosphorylation of Pti1K96N and AvrPtoB. Pto-FLAG, ptoD164N-FLAG, ptoS198A-FLAG, ptoT199A-FLAG, and ptoS198A/T199A-FLAG proteins were transiently expressed in N. benthamiana and immunoprecipitated with anti-FLAG M2 affinity matrix. pti1K96N-His and GST-AvrPtoB substrates were expressed and purified from Escherichia coli. Coomassie Brilliant Blue (CBB) staining of the immunoblot (IB) membranes verified that equal amounts of pti1K96N-His and GST-AvrPtoB were added in each assay. Relative autophosphorylation and transphosphorylation kinase activity was calculated as the ratio between incorporated radioactivity and the amount of immunoprecipitated protein estimated based on CBB staining of the IB membrane and expressed as a percentage of Pto-FLAG relative autophosphorylation or transphosphorylation kinase activity. (TIF) [file ppat.1003123.s006.tif]

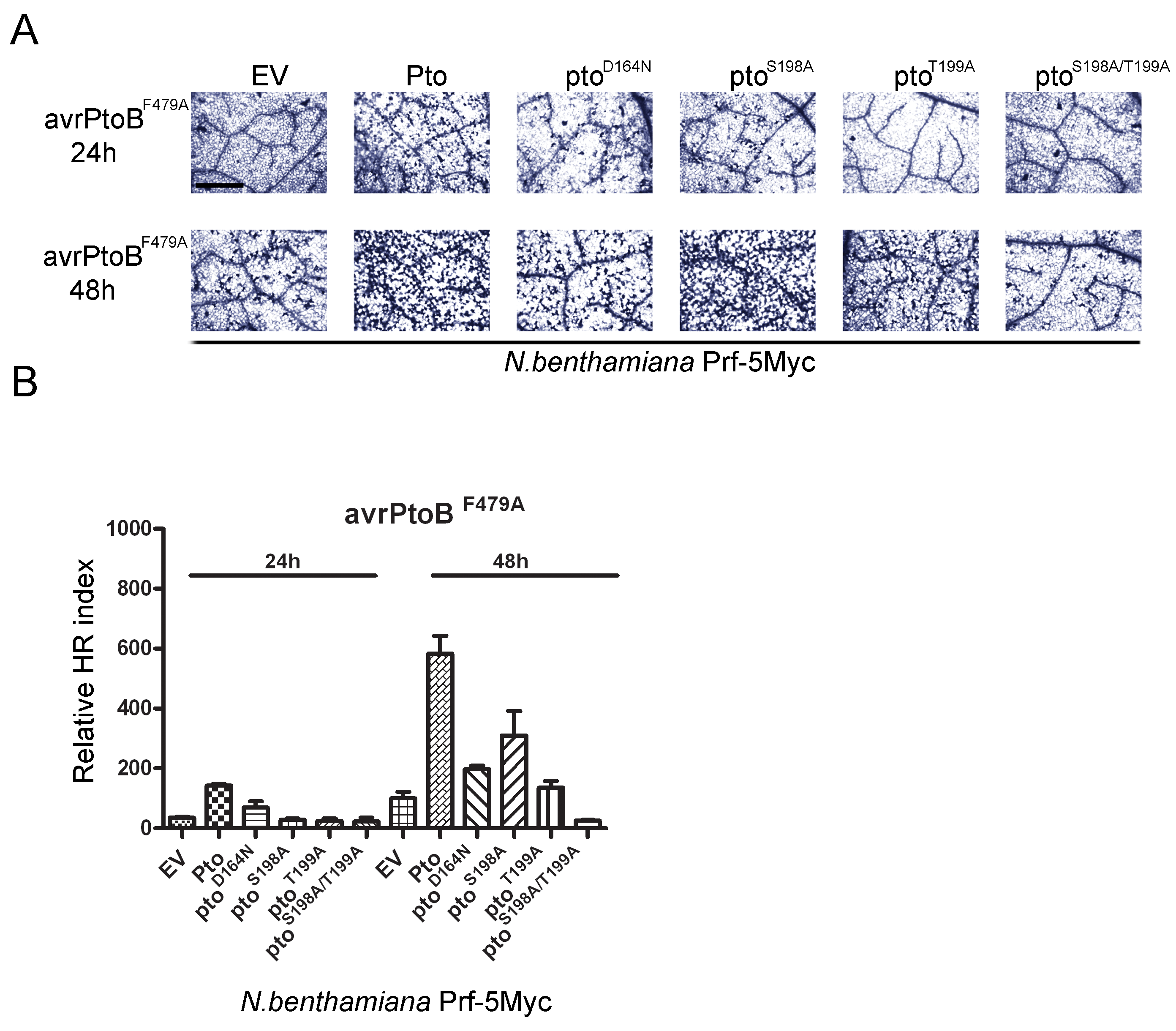

Supplement: Figure S7 — Double phosphorylation on S198 and T199 is necessary for signalling. (A) Trypan blue staining of cell death in N. benthamiana leaves. Pto-FLAG, pto mutant-FLAG and avrPtoBF479A constructs were transiently expressed in stable transgenic ProPrf:Prf-5Myc N. benthamiana leaves as indicated and the tissue was stained 24 or 48 hours post infiltration. The bar indicates 0.5 mm. Dead cells stain dark blue in this qualitative assay. Each row is derived from a single leaf, within which relative amounts of cell death were comparable, and is representative of three replicates. (B) Relative Hypersensitive Response (HR) index. Images of trypan blue staining of cell death in N. benthamiana leaves from three independent experiments were used to estimate the Relative HR index. The proteins were transiently expressed as indicated and pictures were taken as indicated in A. Each graph is derived from three leaves, within which relative amounts of cell death were comparable. Error bars are standard deviation. (TIF) [file ppat.1003123.s007.tif]

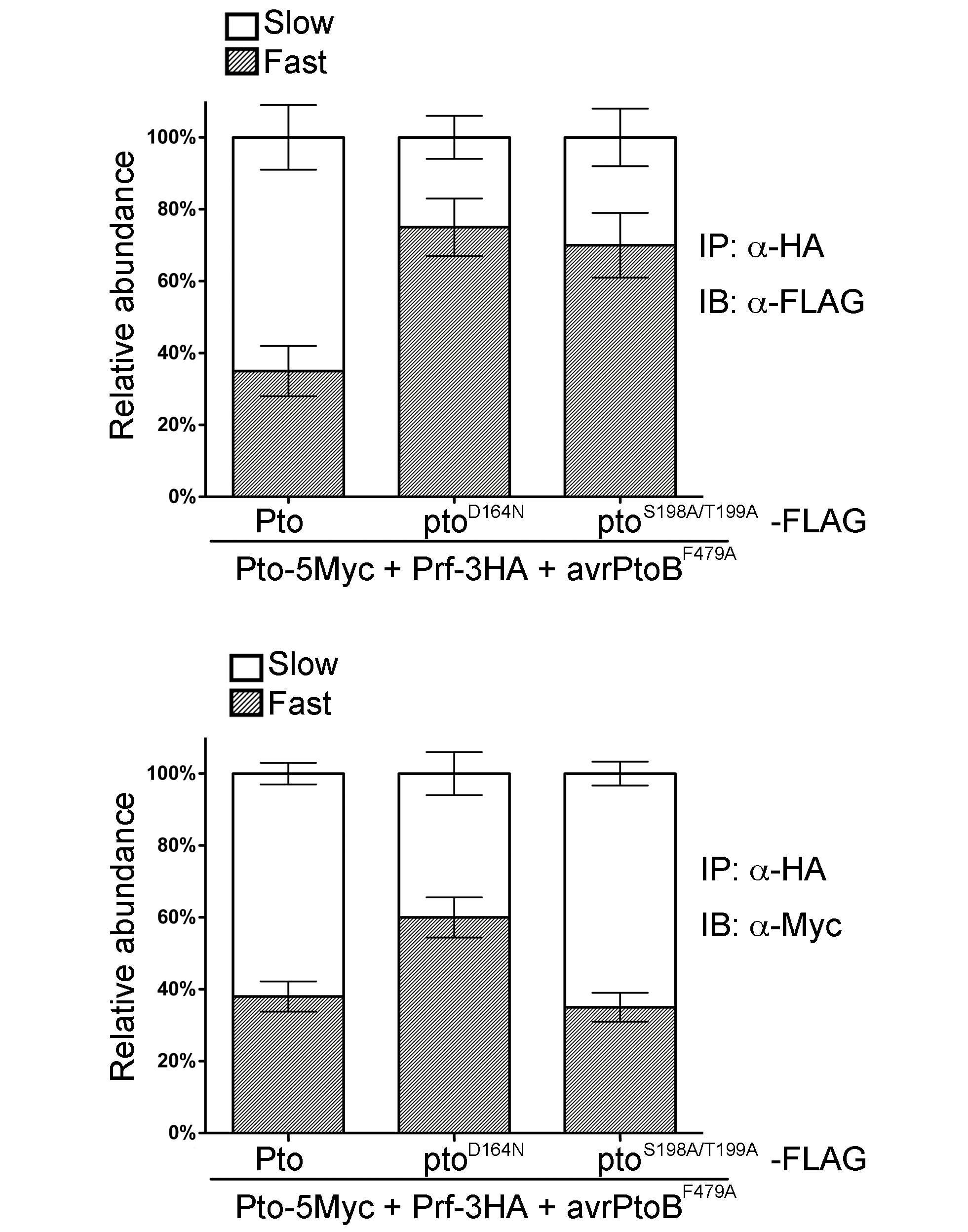

Supplement: Figure S8 — The slower migrating from of Pto was suppressed in trans by ptoD164N, but not by ptoS189A/T199A. Prf-3HA, Pto-5Myc, Pto-FLAG, ptoD164N-FLAG, ptoS189A/T199A -FLAG and avrPtoBF479A constructs were transiently expressed in wild-type N. benthamiana as indicated. The E3 ligase mutant avrPtoBF479A was used instead of wild-type AvrPtoB to avoid ptoD164N degradation. Prf-3HA was immunoprecipitated (IP) using anti-HA antibodies. The relative abundance of slow- and fast-migrating forms of Pto-FLAG, ptoD164N-FLAG, ptoS189A/T199A -FLAG were quantified using anti-FLAG immunoblots (IB) (upper graph). Anti-Myc immunoblots (lower graph) were used for quantification of slow- and fast-migrating forms of Pto-5Myc in the presence of different Pto variants. Quantity One, Bio-Rad (adjusted volume = [CNT*mm2] data counts/mm2) was used for quantification of anti-FLAG and anti-Myc immunoblots. Error bars are standard deviation of relative abundance between the same samples in independent immunoblots, probed with the anti-FLAG or anti-Myc antibodies. (TIF) [file ppat.1003123.s008.tif]

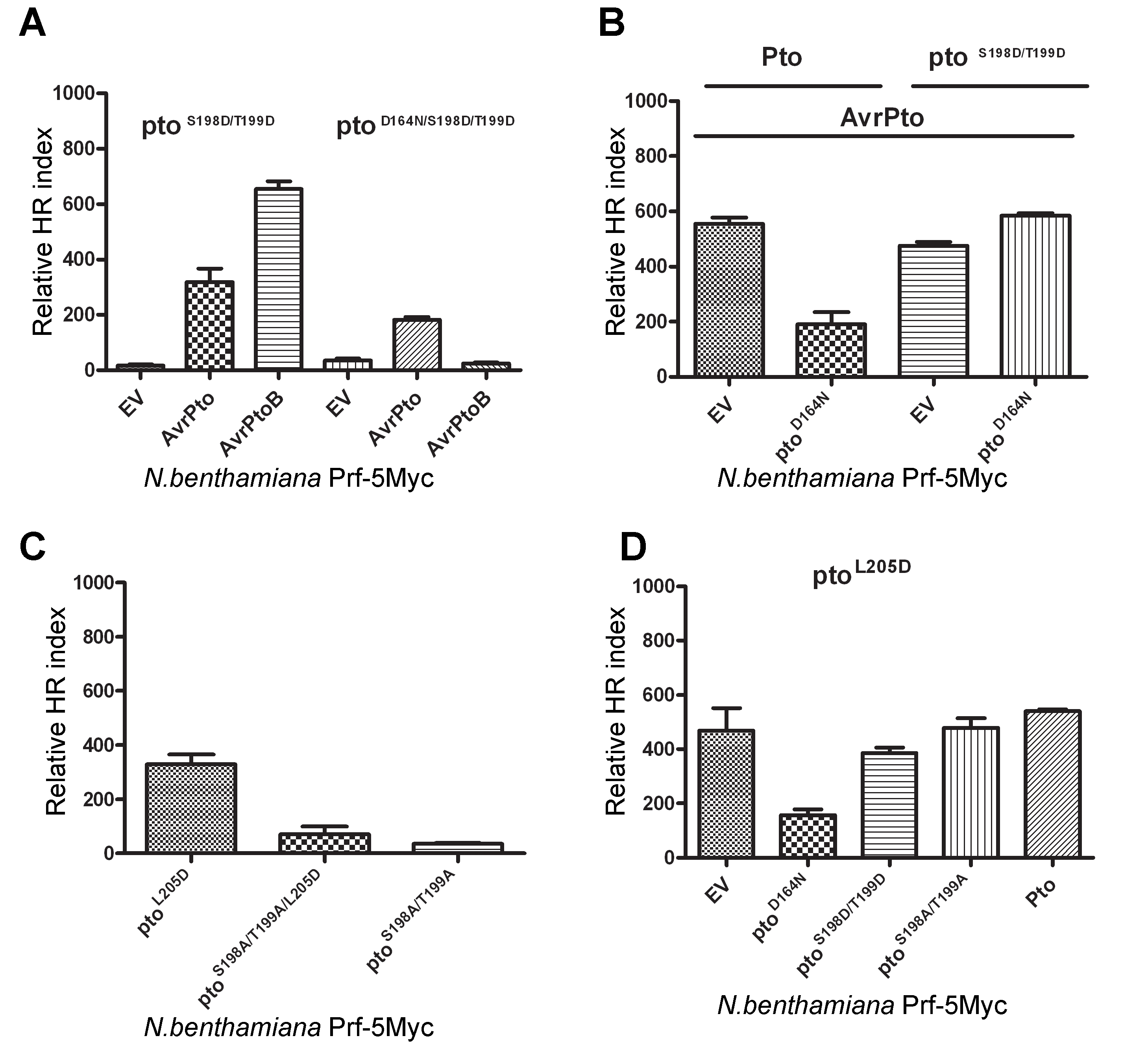

Supplement: Figure S9 — Transphosphorylation is required for induction of cell death. (A,B,C,D) Relative Hypersensitive Response (HR) index was estimated from trypan blue staining of cell death in N. benthamiana leaves based on three independent experiments. Cell death stains dark blue in this qualitative assay and was estimated using ImageJ. The proteins were transiently expressed as indicated and pictures were taken two days post infiltration. Representative pictures are in Figure 4. Each graph is derived from three leaves, within which relative amounts of cell death were comparable. Error bars are standard deviation. (TIF) [file ppat.1003123.s009.tif]

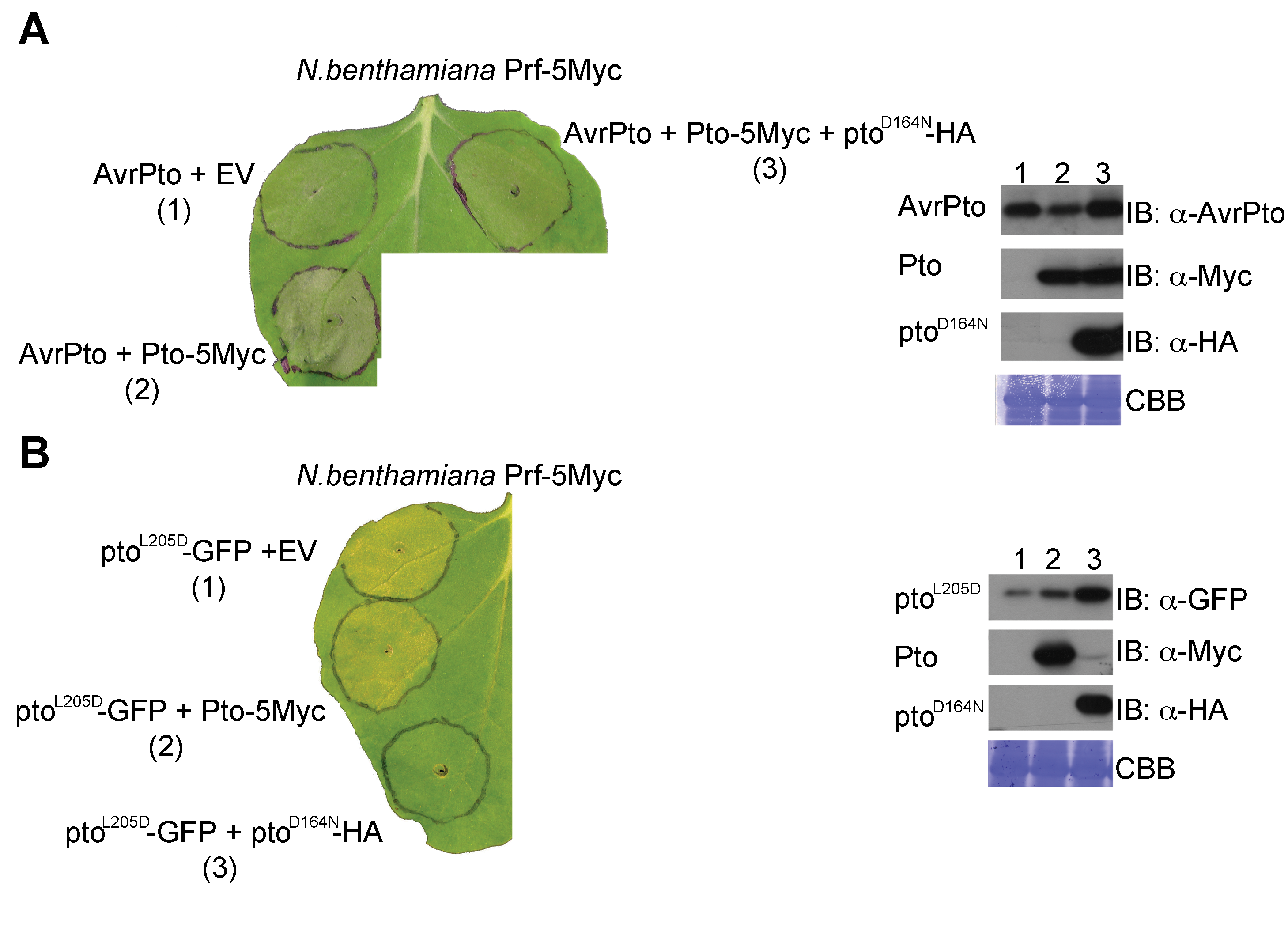

Supplement: Figure S10 — The kinase inactive ptoD164N compromises hypersensitive cell death response (HR) in trans . (A). The Hypersensitive Response (HR) triggered by AvrPto recognition is compromised by kinase-inactive ptoD164N. The indicated AvrPto, Pto-5Myc and ptoD164N-HA constructs were transiently expressed in stable transgenic ProPrf:Prf-5Myc N. benthamiana leaves. The picture was taken at three days post infiltration. Protein expression was confirmed by immunoblots (IB) with the antibodies indicated on the right. Coomassie Brilliant Blue (CBB) staining of the IB membrane verified equal protein loading. The experiment was repeated several times and typical results are shown. (B) The cell death phenotype triggered by the constitutive gain-of-function (CGF) mutant ptoL205D is compromised by the co-expression of kinase-inactive ptoD164N. The indicated ptoL205D-GFP, Pto-5Myc and ptoD164N-HA constructs were transiently expressed in stable transgenic ProPrf:Prf-5Myc N. benthamiana leaves. The picture was taken at three days post infiltration. Protein expression was confirmed by immunoblots with the antibodies indicated on the right. CBB staining of the IB membrane verified equal protein loading. The experiment was repeated several times and typical results are shown. (TIF) [file ppat.1003123.s010.tif]
