# Supplementary material for: The Tomato Prf Complex Is a Molecular Trap for Bacterial Effectors Based on Pto Transphosphorylation
Source: PLoS Pathog. 2013 Jan 31;9(1):e1003123. doi: 10.1371/journal.ppat.1003123 (PMC3561153; doi:10.1371/journal.ppat.1003123)
Supplement: Table S1 — Double phosphorylation of Pto peptide 187–202 and 1888-202 upon activation of signalling. (DOCX) [file ppat.1003123.s011.docx]

**Table S1. Double phosphorylation of Pto peptide 187-202 and 1888-202 upon activation of signalling.**

|  |  |  |  |  |
| --- | --- | --- | --- | --- |
|  | Prf | | | prf^D1416V^ |
|  | EV  (2dpi) | AvrPto (2dpi) | AvrPtoB (2dpi) | EV  (2dpi) |
| Pto peptide 188-202 |  |  |  |  |
| GTELDQTHL[_p_S^198^]TVVK | 0 | 1 | 1 | 2 |
| GTELDQTHLS [_p_T^199^]VVK | 0 | 0 | 0 | 0 |
| **GTELDQTHL[_p_S^198^][_p_T^199^]VVK** | 0 | 1 | 1 | 0 |
| GTELDQTHLSTVVK | 4 | 4 | 8 | 4 |
| Pto peptide 187-202 | | | | |
| KGTELDQTHL[_p_S^198^]TVVK | 3 | 2 | 1 | 3 |
| KGTELDQTHLS [_p_T^199^]VVK | 0 | 0 | 2 | 0 |
| **KGTELDQTHL[_p_S^198^][_p_T^199^]VVK** | 0 | 1 | 0 | 1 |
| KGTELDQ[_p_T^195^]HLS [_p_T^199^]VVK | 0 | 0 | 1 | 0 |
| KGTELDQTHLSTVVK | 4 | 4 | 3 | 9 |
| Pto peptides 187-202 and 188-202 | 11 | 13 | 17 | 19 |
| percentage of peptides with  [_p_S^198^] and [_p_T^199^] | 0% | 15.3% | 5.8% | 5.2% |
| Pto Sequence Coverage | 73% | 79% | 68% | 92% |
| Signalling (HR) | no | yes | yes | yes |

Prf-HA, prf^D1416V^ –HA, Pto-FLAG, AvrPto and AvrPtoB were expressed transiently in *N. benthamiana* under the control of 35S promoter; the total amount of Pto-FLAG was immunoprecipitated using anti-FLAG antibodies. The number of peptides identified with 0, 1, and 2 phosphorylation events is indicated.
