# Supplementary material for: The Tomato Prf Complex Is a Molecular Trap for Bacterial Effectors Based on Pto Transphosphorylation
Source: PLoS Pathog. 2013 Jan 31;9(1):e1003123. doi: 10.1371/journal.ppat.1003123 (PMC3561153; doi:10.1371/journal.ppat.1003123)
Supplement: Table S2 — Double phosphorylation of Pto peptide 187–202 and 188–202 upon activation of signalling. (DOCX) [file ppat.1003123.s012.docx]

|  |  |  |  |
| --- | --- | --- | --- |
|  | Prf | | |
|  | EV  (2dpi) | AvrPto (2dpi) | AvrPtoB (2dpi) |
| Pto peptide 188-202 |  |  |  |
| GTELDQ[_p_T^195^]HLS TVVK | 0 | 1 | 0 |
| GTELDQTHL[_p_S^198^]TVVK | 1 | 2 | 1 |
| GTELDQTHLS [_p_T^199^]VVK | 0 | 0 | 1 |
| G[_p_T^190^]ELDQTHLS[_p_T^199^]VVK | 0 | 1 | 0 |
| **GTELDQTHL[_p_S^198^][_p_T^199^]VVK** | 0 | 0 | 1 |
| GTELDQTHLSTVVK | 9 | 2 | 20 |
| Pto peptide 187-202 | | | |
| KGTELDQ[_p_T^195^]HLS TVVK | 0 | 1 | 0 |
| KGTELDQTHL[_p_S^198^]TVVK | 0 | 1 | 0 |
| **KGTELDQTHL[_p_S^198^][_p_T^199^]VVK** | 0 | 1 | 1 |
| KGTELDQTHLSTVVK | 3 | 1 | 7 |
| Pto peptides 187-202 and 188-202 | 13 | 10 | 31 |
| percentage of peptides with  [_p_S^198^] and [_p_T^199^] | 0% | 10% | 6.4% |
| Pto Sequence Coverage | 62% | 45% | 83% |
| Signalling (HR) | no | yes | yes |

**Table S2. Double phosphorylation of Pto peptide 187-202 and 188-202 upon activation of signalling.**

Prf-HA, Pto-FLAG, AvrPto and AvrPtoB were expressed transiently in *N. benthamiana* under the control of 35S promoter; the total amount of Pto-FLAG was immunoprecipitated using anti-FLAG antibodies. The number of peptides identified with 0, 1, and 2 phosphorylation events is indicated.
