# Supplementary material for: The Tomato Prf Complex Is a Molecular Trap for Bacterial Effectors Based on Pto Transphosphorylation
Source: PLoS Pathog. 2013 Jan 31;9(1):e1003123. doi: 10.1371/journal.ppat.1003123 (PMC3561153; doi:10.1371/journal.ppat.1003123)
Supplement: Table S3 — Summary of the different complex combinations examined, and their signalling outcomes. (DOCX) [file ppat.1003123.s013.docx]

|  | ***N.benthamiana***  **Pto**  **active kinase** | **Tomato**  **Pto**  **active kinase** | **Tomato**  **pto^D164N^**  **inactive kinase** | **Tomato**  **pto^S198A^**  **active kinase** | **Tomato**  **pto^T199A^**  **active kinase** | **Tomato**  **pto^S198A/T199A^**  **active kinase** | **Tomato pto^S198D/T199D^**  **active kinase** | **Tomato pto^D164N/S198D/T199D^**  **inactive kinase** | **Tomato**  **pto^L205D^**  **inactive kinase** | **Tomato pto^S198A/T199A/L205D^**  **inactive kinase** |
| --- | --- | --- | --- | --- | --- | --- | --- | --- | --- | --- |
| ***N.benthamiana* Pto**  **active kinase** | ***no*** | ***yes*** | ***no***  ***yes avrPtoB^F479A^*** | ***yes*** | ***yes*** | ***no***  ***no avrPtoB^F479A^*** | ***yes*** | ***yes (AvrPto)***  ***no (AvrPtoB)*** | ***yes*** | ***no*** |
| **Tomato**  **Pto**  **active kinase** | ***yes*** | ***yes*** | ***no*** |  |  |  |  |  | ***yes*** |  |
| **Tomato**  **pto^D164N^**  **inactive kinase** | ***no***  ***yes avrPtoB^F479A^*** | ***no*** | ***no*** |  |  |  |  |  | ***no*** |  |
| **Tomato**  **pto^S198A^**  **active kinase** | ***yes*** |  |  | ***yes*** |  |  |  |  |  |  |
| **Tomato**  **pto^T199A^**  **active kinase** | ***yes*** |  |  |  | ***yes*** |  |  |  |  |  |
| **Tomato**  **pto^S198A/T199A^**  **active kinase** | ***no***  ***no avrPtoB^F479A^*** |  |  |  |  | ***no*** |  |  | ***yes*** |  |
| **Tomato**  **pto^S198D/T199D^**  **active kinase** | ***yes*** |  |  |  |  |  | ***yes*** |  | ***yes*** |  |
| **Tomato pto^D164N/S198D/T199D^**  **inactive kinase** | ***yes (AvrPto)***  ***no (AvrPtoB)*** |  |  |  |  |  |  | ***yes (AvrPto)***  ***no (AvrPtoB)*** |  |  |
| **Tomato**  **pto^L205D^**  **inactive kinase** | ***yes*** | ***yes*** | ***no*** |  |  | ***yes*** | ***yes*** |  |  |  |
| **Tomato pto^S198A/T199A/L205D^**  **inactive kinase** | ***no*** |  |  |  |  |  |  |  |  | ***no*** |

**Table S3. Summary of the different complex combinations examined, and their signalling outcomes.**

Reconstitution of the tomato complex in *N.benthamiana* by expression of tomato Prf and different variants of tomato Pto. *Yes* or *no* is for signalling in the presence of AvrPto and AvrPtoB or by constitutive gain-of-function mutants of Pto.
